# Treatment of idiopathic pulmonary fibrosis and progressive pulmonary fibrosis: A position statement from the Thoracic Society of Australia and New Zealand 2023 revision

**DOI:** 10.1111/resp.14656

**Published:** 2024-01-11

**Authors:** John A. Mackintosh, Gregory Keir, Lauren K. Troy, Anne E. Holland, Christopher Grainge, Daniel C. Chambers, Debra Sandford, Helen E. Jo, Ian Glaspole, Margaret Wilsher, Nicole S. L. Goh, Paul N. Reynolds, Sally Chapman, Steven E. Mutsaers, Sally de Boer, Susanne Webster, Yuben Moodley, Tamera J. Corte

**Affiliations:** ^1^ Department of Respiratory Medicine The Prince Charles Hospital Brisbane Queensland Australia; ^2^ Centre of Research Excellence in Pulmonary Fibrosis Camperdown New South Wales Australia; ^3^ Department of Respiratory Medicine Princess Alexandra Hospital Brisbane Queensland Australia; ^4^ Department of Respiratory and Sleep Medicine Royal Prince Alfred Hospital Camperdown New South Wales Australia; ^5^ University of Sydney Sydney New South Wales Australia; ^6^ Department of Physiotherapy The Alfred Hospital Melbourne Victoria Australia; ^7^ Department of Respiratory Research@Alfred Central Clinical School, Monash University Melbourne Victoria Australia; ^8^ Department of Respiratory Medicine John Hunter Hospital Newcastle New South Wales Australia; ^9^ Department of Thoracic Medicine Central Adelaide Local Health Network Adelaide South Australia Australia; ^10^ University of Adelaide Adelaide South Australia Australia; ^11^ Department of Respiratory Medicine The Alfred Hospital Melbourne Victoria Australia; ^12^ Department of Respiratory Medicine Te Toka Tumai Auckland Auckland New Zealand; ^13^ Department of Respiratory Medicine Austin Hospital Melbourne Victoria Australia; ^14^ Institute for Breathing and Sleep Melbourne Victoria Australia; ^15^ University of Melbourne Melbourne Victoria Australia; ^16^ Institute for Respiratory Health, University of Western Australia Nedlands Western Australia Australia; ^17^ Department of Respiratory Medicine Fiona Stanley Hospital Murdoch Western Australia Australia

**Keywords:** idiopathic pulmonary fibrosis, interstitial lung disease, progressive pulmonary fibrosis, pulmonary fibrosis, treatment

## Abstract

Idiopathic pulmonary fibrosis (IPF) is a progressive disease leading to significant morbidity and mortality. In 2017 the Thoracic Society of Australia and New Zealand (TSANZ) and Lung Foundation Australia (LFA) published a position statement on the treatment of IPF. Since that time, subsidized anti‐fibrotic therapy in the form of pirfenidone and nintedanib is now available in both Australia and New Zealand. More recently, evidence has been published in support of nintedanib for non‐IPF progressive pulmonary fibrosis (PPF). Additionally, there have been numerous publications relating to the non‐pharmacologic management of IPF and PPF. This 2023 update to the position statement for treatment of IPF summarizes developments since 2017 and reaffirms the importance of a multi‐faceted approach to the management of IPF and progressive pulmonary fibrosis.

## INTRODUCTION

In 2017 a group of Australian and New Zealand authors, on behalf of the Thoracic Society of Australia and New Zealand (TSANZ) and Lung Foundation Australia (LFA), collaborated on a position statement on the treatment of Idiopathic Pulmonary Fibrosis (IPF).^1^ This paper was published contemporaneously with access to subsidized anti‐fibrotic therapy, for patients with IPF. Since 2017 significant scientific advances have been made in the field of interstitial lung disease (ILD), resulting in important changes in clinical practice including the recent subsidized listing of nintedanib for non‐IPF progressive pulmonary fibrosis (PPF) in Australia and a new Clinical Practice Guideline from the ATS/ERS/JRS/ALAT for both IPF and PPF.^2^ In light of these developments, the publication of numerous seminal papers, and following 5 years of accumulated local clinical experience in the anti‐fibrotic era, it was deemed appropriate timing for an update to the Treatment of IPF Position Statement.

While there have been significant advances in our knowledge of IPF since 2017, the disease remains progressive despite anti‐fibrotic therapy, and ultimately results in death in the majority of cases. Recent Australian data demonstrates overall crude estimates of incidence, prevalence and mortality for IPF of 10.4, 32.6 and 5.9 per 100,000 population, respectively.^3^ Meta‐analysis reveals a dismal five‐year cumulative survival rate of 45.6%.^4^ In this position statement, we will focus on updates to the approach to IPF management since 2017.

This update also canvases treatment of non‐IPF PPF. Recent clinical trials have suggested a broader benefit for anti‐fibrotic medications in non‐IPF fibrotic diseases demonstrating PPF disease behaviour. The efficacy of nintedanib demonstrated in the INBUILD study^5^ has now been recognized by the Australian Pharmaceutical Benefits Scheme (PBS) with its listing across a range of non‐IPF disease states. The pharmacological landscape for fibrotic ILD management is now more complicated than ever. There is also growing evidence and literature on the non‐pharmacological aspects of management. PPF, as a very recent entity, unfortunately does not have the same weight of evidence as for IPF, with management outside of anti‐fibrotic therapy adopted from IPF or the specific underlying ILD. This update aims to provide guidance to clinicians, to deliver the most effective care to those living with IPF and PPF.

## METHODS

This position paper provides an update to the paper which was published in 2017.^1^ The contents of the 2017 position paper are not covered in detail in this update, which focuses on new developments since the 2017 publication, up to and including August 2023. Like the previous paper, the intention is to provide highlights on important developments in the treatment of IPF, and now PPF, as they pertain to Australia and New Zealand. The position paper does not represent a guideline, but is intended to enhance the knowledge of clinicians involved in the management of this disease. The expert panel was comprised of 14 respiratory physicians, 1 respiratory scientist, 1 physiotherapist, 1 respiratory nurse and 1 psychologist, who contributed to all stages of the planning and writing process, with representation from both Australia and New Zealand. Authors were allocated specific sections of the paper to complete based on their specific expertise or interests. Authors were encouraged to focus on literature published since 2017. All members of the panel reviewed the compiled sections and approved the final manuscript. This work received no commercial sponsorship, and the authors completed this work on an honorary basis. The position paper has been reviewed by the consumer group affiliated with the Centre of Research Excellence in Pulmonary Fibrosis and is endorsed by the Thoracic Society of Australia and New Zealand and will be disseminated via publication in *Respirology*. This document will be reviewed within a maximum of 5 years.

## PHARMACOLOGICAL THERAPY

### Anti‐fibrotic therapy

Two medications, nintedanib and pirfenidone, are effective in slowing the decline in lung function in IPF and are recommended for IPF in multinational clinical practice guidelines.^6^ These medications are licensed for the treatment of mild to moderate IPF (according to lung function criteria) in both Australia and New Zealand (Table 1). Both medications have been shown in randomized, double blind placebo controlled studies to slow the rate of decline in forced vital capacity (FVC) by approximately 50%.^7^, ^8^ Pooled trial data and post hoc analyses suggest both drugs reduce the incidence of acute exacerbation.^9^ Efficacy appears to be similar for both medications. A comprehensive description of the clinical trial data can be found in the previous version of this position statement.^1^

**TABLE 1:** Anti‐Fibrotic (nintedanib and pirfenidone) Subsidized Prescribing Conditions for Idiopathic Pulmonary Fibrosis in Australia and New Zealand as of May 2023.

	Australia	New Zealand
Diagnosis	Confirmed by a multidisciplinary meeting	Confirmed by a multidisciplinary meeting
Physiology	FVC ≥50% predicted	FVC between 50% and 90% predicted
	DLCO ≥30% predicted	
	FEV_1_/FVC >0.7	
HRCT	Consistent with IPF within previous 12 months	
Stopping rule	Nil	≥10% decline in FVC% predicted within a 12 month period

Abbreviations: DLCO, diffusing capacity for carbon monoxide; FEV1, forced expiratory volume in 1 s; FVC, forced vital capacity; HRCT, high‐resolution computed tomography; IPF, idiopathic pulmonary fibrosis.

In the randomized controlled trials of nintedanib, health related quality of life declined over 12 months (below the minimal important difference), but there was statistically significant less decline in the nintedanib group.^10^, ^11^ While health related quality of life was not directly assessed in the pirfenidone trials, dyspnoea, which contributes significantly to quality of life impairment was. Participants in both arms of the studies demonstrated increased levels of dyspnoea over the 12 month trial period, however, the proportion with a significant increase was lower in those on pirfenidone.^10^, ^12^ A very small observational study of pirfenidone has suggested an improvement in objective 24 h cough counts,^13^ however, there appears to be no improvement in cough with nintedanib.^14^ Combined data from the pirfenidone trials demonstrated a reduced risk of respiratory‐related hospitalization.^15^

While the initial anti‐fibrotic clinical trials were not designed or powered to detect survival benefit, in a prespecified survival analysis including data from the CAPACITY and ASCEND studies, pirfenidone reduced the risk of death from any cause at 1 year by 48% (hazard ratio [HR] 0.52; 95% confidence interval [CI] 0.31–0.87; *p* = 0.01) and reduced the risk of death from IPF at 1 year by 68% (HR 0.32; 95% CI 0.14–0.76; *p* = 0.006), when compared to placebo.^7^ A recent meta‐analysis comprising only randomized clinical trials confirmed a benefit on 12 month all‐cause mortality for pirfenidone (HR 0.5, 95%CI 0.31–0.83).^16^ Combined data for the impact of nintedanib on 12 month all‐cause mortality demonstrated a HR 0.69 (95%CI 0.44–1.07).^16^

The longer term prognostic impact of anti‐fibrotic therapy, beyond the 12‐month duration of most clinical trials, has been demonstrated in observational data which has generally indicated benefit (RR 0.55 (95%CI 0.45–0.66)).^17^ A number of national IPF registries, including Australia's IPF registry, have also reported survival benefit with anti‐fibrotic therapies compared to historical untreated cohorts.^18^, ^19^, ^20^ It should be noted that this observational data is, by definition, associated with a number of biases.^21^ Unfortunately, robust clinical trial data to confirm a long‐term survival benefit of anti‐fibrotic therapy are lacking.

It has been postulated that combination therapy with pirfenidone and nintedanib may result in greater attenuation of FVC decline, than either alone, as these treatments have different mechanisms of action. While several studies have demonstrated acceptable safety and tolerability (albeit with slightly higher rates of nausea and vomiting), superior efficacy with combination anti‐fibrotic therapy has not been proven.^14^, ^15^, ^16^ Combination anti‐fibrotic therapy is not currently recommended outside of clinical trials, nor is it subsidized in Australia or New Zealand.

#### Adverse events

Adverse events (AEs) with both pirfenidone and nintedanib are relatively frequent, although rates of discontinuation due to AEs were low in the clinical trials. Combined data from the pirfenidone studies report the most common AEs to be nausea (in 37.6% of patients), diarrhoea (28.1%), dyspepsia (18.4%), vomiting (15.9%) and photosensitive rash (25%), although these were considered generally mild and without significant clinical consequences.^22^ Real world treatment data have been reported in the PASSPORT study,^23^ a multi‐centre prospective, post‐authorisation registry which followed 1009 IPF patients for 2 years after initiating treatment with pirfenidone. Overall, 73.4% of patients experienced AEs related to pirfenidone, most commonly nausea (20.6%) and fatigue (18.5%). Photosensitivity reaction occurred in 5.8% of patients. All patients should be counselled regarding the avoidance of direct sunlight exposure and sun protection measures. AEs leading to pirfenidone discontinuation occurred in 28.7% of patients after a median of 99.5 days.

In the nintedanib studies, the most frequent AE was diarrhoea (with rates of approximately 60% compared to approximately 18% with placebo), which resulted in cessation of the drug in <5% of patients.^24^ Other common nintedanib related AEs included nausea, vomiting and decreased appetite, occurring in 8%–25% of patients. Long‐term safety and tolerability of nintedanib have been evaluated in an open‐label extension INPULSIS‐ON study,^25^ which included 734 patients with a median exposure time to nintedanib treatment of 44.7 months (range 11.9–68.3). Diarrhoea was the most frequent AE, with 60–70 events per 100 patient exposure‐years reported. Fifty‐one patients (6.9%) permanently discontinued nintedanib because of diarrhoea.

Serious liver function abnormalities (ALT/AST greater than three times the upper limit of normal, and/or elevated bilirubin greater than two times the upper limit of normal) were observed in 3.7% of those taking pirfenidone in a pooled analysis of all trials.^22^ These abnormalities were typically observed within the first 6 months of treatment. Serious ALT/AST elevations occurred in 5% of nintedanib treated patients in the INPULSIS trials, with bilirubin elevations greater than two times the upper limit of normal in 0.5%, the majority of events occurring within the first 3 months of treatment.^24^ Reversal of the liver function derangement occurred with dose reduction/interruption of the anti‐fibrotic. Both anti‐fibrotics are contraindicated in severe hepatic impairment. Cautious monitoring of liver function is required after commencement of treatment as specified in the product information. If liver function derangement precludes continued use of one anti‐fibrotic, the other agent should be considered as intolerance of one does not predict intolerance to the other.

Cardiovascular disease and its risk factors are common in patients with IPF. Concerns have previously been raised of the cardiovascular risk associated with nintedanib.^26^ Pooled data from INPULSIS and TOMORROW, which comprised many patients with elevated cardiovascular risk, demonstrated a low incidence of major adverse cardiovascular events (MACE), which was similar between nintedanib (incidence rate for high cardiovascular risk 3.88 (95%CI 2.58–5.84) per 100 patient‐years) and placebo (incidence rate for high cardiovascular risk 3.49 (95%CI 2.10–5.79) per 100 patient‐years).^27^ Analysis of a global pharmacovigilance database, comprising 60,107 patient years identified an event rate of MACE that was lower than in the INPULSIS and TOMORROW trials, and lower than that reported for IPF patients not treated with nintedanib.^28^, ^29^ Cardiovascular risk should be considered when prescribing an anti‐fibrotic, however, the impact of nintedanib on this risk is unlikely to be significant in most instances.

The mechanism of action of nintedanib as an inhibitor of VEGF^30^ suggests a potential association with bleeding. In the nintedanib clinical trials, non‐serious epistaxis was the most common bleeding complication occurring at an increased rate compared to placebo,^26^ with no increase in serious bleeding events; notably, patients receiving full dose anticoagulation or with an inherited predisposition to bleeding were excluded from the trials. European registry data reveals an overall low incidence of bleeding in people with IPF treated with anti‐fibrotics (0.29%), although pirfenidone was preferred in people on anticoagulants.^31^ There were seven bleeding events among the 673 nintedanib treated individuals, only two of which were co‐prescribed an antiplatelet or anticoagulant. The decision to commence nintedanib in someone with a known risk for bleeding should follow careful evaluation of the benefit to risk in discussion with the patient, with a decision to proceed if the beneficial effect of anti‐fibrotic therapy is likely to outweigh the potential bleeding risk.

#### Management of AEs

Several strategies are available to help patients to manage AEs associated with anti‐fibrotic use. It is recommended that both pirfenidone and nintedanib are taken with meals to reduce the risk of gastrointestinal adverse effects. For nintedanib‐associated diarrhoea, dietary modification (including a low fibre diet and avoidance of spicy foods) coupled with the use of anti‐diarrhoeal medication (e.g., loperamide) can be helpful. The risk of pirfenidone associated photosensitivity can be minimized by avoiding exposure to direct sunlight (including a broad brimmed hat and long‐sleeve shirts) and use of highly protective (50+) sunscreen. Additional information for patients and carers regarding AE management can be found at the Lung Foundation Australia website (Anti‐fibrotic Treatments for IPF—Lung Foundation Australia). Finally, drug interactions should be considered before prescribing anti‐fibrotics. Pirfenidone is metabolized by CYP1A2, with inhibitors of this enzyme such as ciprofloxacin and fluvoxamine best avoided or otherwise associated with a substantial dose reduction and careful monitoring. Nintedanib is less prone to drug interactions, however, concentrations may be impacted by strong p‐glycoprotein inducers or inhibitors.

Temporary dose modifications, including dose reductions or interruptions, may also help in the management of AEs. In a pooled analysis from pirfenidone studies, a similar effect on FVC preservation was noted in patients who received either >90% or <90% of the expected pirfenidone treatment dose.^8^ Similar data have been reported for nintedanib.^25^ While these results suggest that a lower dose of pirfenidone or nintedanib remain effective in slowing FVC decline in IPF, the minimal effective dose is not clear. In patients considered to be at higher risk of intolerance by virtue of older age, co‐morbidities and other factors, some clinicians consider starting at a lower dose of nintedanib or with a more prolonged pirfenidone dose escalation, although there are no data to support this approach.

#### Treatment of IPF patients with severe disease

There is no universal consensus on how to define disease severity of patients with IPF, with several different methods proposed. Real life and post hoc trial data suggest patients with mild disease respond to anti‐fibrotic medication as well as patients with moderate disease, although funding authorities may still restrict access on the basis of lung function parameters (Table 1).^9^, ^20^, ^32^, ^33^ There is significant variation between predictive reference equations for FVC% and DLCO%, which might impact on eligibility for anti‐fibrotic therapy.^34^

Although most clinical trials have excluded patients with severe IPF based on physiologic criteria, post hoc analysis of data from INPULSIS and INSTAGE trials shows that nintedanib had a similar effect on FVC decline in patients with IPF irrespective of severity of gas exchange impairment at baseline.^33^ Similarly, post hoc analyses of ASCEND and RECAP trial data indicate benefit with pirfenidone across multiple domains in patients with FVC <50% and/or DLCO <35%.^35^, ^36^ Real life data from small cohorts suggests that nintedanib and pirfenidone slow the rate of decline of physiologic parameters in patients with FVC less than 50% predicted and or GAP II/III.^37^, ^38^, ^39^, ^40^

The reported rate of AEs is similar irrespective of disease severity, although patients with more severe disease have a higher discontinuation rate. On the basis of the above data, while access to anti‐fibrotic therapy may not be subsidized for patients with severe lung function impairment at baseline, the decision to continue anti‐fibrotic therapy should balance any anticipated benefit against potential negative impacts of anti‐fibrotic therapy (e.g., side effects) on quality of life.

#### Treatment with low diagnostic certainty

While this position paper focuses on the management of IPF, rather than its diagnosis, it is important to recognize that the certainty or confidence level of an IPF diagnosis may vary depending on clinical findings. The current ATS/ERS/JRS/ALAT IPF guideline update carries forward previous recommendations on when to consider an IPF diagnosis in the context of varying high‐resolution chest computed tomography (HRCT) and histological patterns.^2^ It is outside the scope of this paper to provide further detail on the weighting of IPF diagnostic confidence.^41^ However, a ‘provisional’ or ‘working diagnosis’ of IPF from multi‐disciplinary discussion,^42^, ^43^, ^44^ should be viewed as conducive to consideration of that patient for anti‐fibrotic therapy. Supporting this pragmatic approach is the INPULSIS trial, where patients without honeycombing present on HRCT and absent confirmatory histology (i.e., possible usual interstitial pneumonia (UIP)), had a similar response to nintedanib as those with definite UIP.^45^ Australian IPF Registry data also support this approach, showing that patients not meeting guideline criteria for a confident IPF diagnosis, had identical disease behaviour in terms of lung function decline and survival to those who did meet the IPF diagnostic criteria.^46^ In the setting of a ‘low confidence’ IPF diagnosis (i.e., 51%–69% certainty) clinicians should remain vigilant for the development of features that might indicate an alternative diagnosis, potentially enabling other treatment options that could stabilize or even improve disease.

#### When to start, switch or stop anti‐fibrotic medication

The decision to start treatment needs to be individualized and should always be in the context of a discussion with the patient and their family, with consideration of comorbidities, age and awareness of potential toxicity. Given that both anti‐fibrotics demonstrate efficacy in early/mild disease, all patients should be considered for anti‐fibrotic therapy at IPF diagnosis. Funding is not currently available in New Zealand for mild disease with FVC greater than 90%, whereas no such limit applies in Australia.

There is often hesitancy to consider anti‐fibrotic treatment in older patients for fear of intolerance and nihilism surrounding benefit. A pooled analysis of five nintedanib clinical trials demonstrated similar slowing of FVC decline in IPF patients older than 75 years and in those with multiple co‐morbidities.^47^ Rates of AEs leading to treatment discontinuation were higher in those over 75 years and in those with multiple co‐morbidities, however, the majority of patients in these categories were able to tolerate nintedanib. Older patients and those with co‐morbidities require careful informed decision making before commencement of anti‐fibrotic treatment and proactive management of AEs.

The most common reasons for patients discontinuing anti‐fibrotic medication are disease progression and treatment toxicity.^48^ Many patients tolerate the alternative drug in the event of initial treatment toxicity. In New Zealand and Australia, funding authorities permit treatment switch between anti‐fibrotic agents for toxicity. There is no evidence that switching to an alternative anti‐fibrotic favourably impacts disease progression. Significant disease progression in an individual otherwise tolerating their current anti‐fibrotic agent should not necessarily prompt conversion to the other agent. The current anti‐fibrotic agents slow but do not stop disease progression. Switching treatments often leads to time off therapy, and potentially exposes patients to new intolerances.

- *Anti‐fibrotic therapy to slow IPF disease progression should be considered at IPF diagnosis in all patients*.
- *Some data suggest that anti‐fibrotic medications may reduce frequency of acute exacerbations and improve survival in patients with IPF*.
- *The decision to start, and to stop, treatment with an anti‐fibrotic should be individualized, and is influenced by disease stage, patient preferences, consideration of comorbidities and risk of toxicity, plus local funding restrictions*.
- *There is no evidence that switching to the alternative anti‐fibrotic is of benefit in those with progressive disease*.
- *Adverse effects are not uncommon, but generally manageable and only occasionally lead to discontinuation*.
- *Combination anti‐fibrotic therapy is not currently recommended*.

### Anti‐reflux therapy

The ATS/ERS had previously conditionally recommended medical treatment of asymptomatic gastro‐oesophageal reflux in the 2011^49^ and 2015^6^ IPF guidelines. In the latest iteration of the guidelines, the recommendation was against the use of anti‐acid pharmacotherapy for the purpose of improving respiratory outcomes.^2^ The predominantly observational evidence has shown varying impacts of gastro‐oesophageal reflux therapy on respiratory outcomes in patients with IPF. Two large post hoc analyses of the pooled data from the pirfenidone^50^ and nintedanib^51^ studies found no benefit of anti‐acid therapy. Concerningly, in the pirfenidone studies, use of anti‐acid therapy was associated with an increased risk of generalized and respiratory infections in patients with more severe IPF (FVC <70%).^50^ In a large UK cohort study, 1852 proton pump inhibitor (PPI) users with IPF were matched to 1852 non‐users, with no differences observed for respiratory‐related hospitalisations, respiratory‐related mortality or all‐cause mortality.^52^ Similarly, no difference in survival or disease progression, regardless of anti‐acid treatment, was observed in the Australian IPF Registry cohort.^53^ In 2018, a meta‐analysis of 13 observational cohort studies, including almost 1500 participants, was published.^54^ Fidler et al. concluded that pharmacologic treatment of gastro‐oesophageal reflux was associated with a reduction in IPF‐related but not all‐cause mortality. However, the evidence was acknowledged to be of low quality.^54^

The WRAP‐IPF study was a randomized controlled trial of laparoscopic anti‐reflux surgery in patients with IPF, with 58 patients assigned to surgical (*n* = 29) or non‐surgical (*n* = 29) arms. No difference in the primary endpoint (rate of change in FVC) was observed between the surgical (−0.05 L (95% CI −0.15 to 0.05)) and non‐surgical (−0.13 L (−0.23 to −0.02)) arms (*p* = 0.28). Acute exacerbation, respiratory‐related hospitalization and death were less common, but not significantly so, in the surgical group, however, the study was underpowered for these endpoints.^55^

The 2022 ATS/ERS/JRS/ALAT IPF and PPF clinical practice guideline made a conditional recommendation against anti‐reflux surgery for the purpose of improving respiratory outcomes in IPF.^2^ A meta‐analysis that accompanied the updated guideline determined that anti‐acid medication did not significantly prevent declines in FVC or 6‐min walk distance or reduce risk of death.^56^ For both anti‐acid pharmacotherapy and surgery there is a need for larger, prospective clinical trials with inclusion stratified by objective measures of gastro‐oesophageal reflux at baseline.

- *There is insufficient evidence to support a beneficial role of anti‐acid pharmacotherapy or surgery for respiratory outcomes in patients with IPF*.
- *Symptomatic gastro‐oesophageal reflux should be approached per clinical guidelines for the general population*.

### Progressive pulmonary fibrosis

#### Definition of progressive pulmonary fibrosis (PPF)

The concept of PPF has evolved in recent years, as the importance of disease behaviour and outcomes have been acknowledged across the spectrum of ILDs.^57^, ^58^ Many non‐IPF ILD subtypes progress to end‐stage fibrosis, despite adherence to evidence‐based or consensus‐based therapy, highlighting the need to consider multi‐modal treatment strategies.

In the recently published 2022 ATS/ERS/JRS/ALAT IPF guidelines, PPF is adopted as the favoured nomenclature.^2^ It is defined as a patient with ILD of known or unknown aetiology other than IPF with radiological evidence of pulmonary fibrosis and clinical evidence of progression over the preceding 12 months. At least two of three criteria are required: *1. Worsening respiratory symptoms not otherwise explained; 2. Physiological evidence of disease progression, (absolute decline in FVC ≥5% or DLCO ≥10% over 12 months); and 3. Radiological evidence of disease progression, (such as increased traction bronchiectasis, bronchiolectasis, ground glass change with traction bronchiectasis, fine reticulation, reticular abnormality, honeycombing and volume loss)*. Notably, these criteria differ to the requirements for subsidized access to nintedanib in Australia for PPF, which also allow for progression to have been observed over 24 months, having been adopted from the INBUILD trial eligibility criteria for progressive fibrosing ILD (PF‐ILD) (Table 2).

**TABLE 2:** Australian pharmaceutical benefits scheme authority criteria for nintedanib in progressive fibrosing interstitial lung disease (PF‐ILD) and ATS/ERS/JRS/ALAT progressive pulmonary fibrosis criteria (PPF) as of May 2023.

	Australian PBS PF‐ILD criteria	ATS/ERS/JRS/ALAT PPF criteria
Diagnosis	Diagnosis other than IPF through a multi‐disciplinary meeting	Any fibrotic ILD other than IPF
	Not due to reversible causes	
Physiology	FVC ≥45%	
	DLCO ≥30% and ≤ 80%	
	FEV1/FVC >0.7	
HRCT	HRCT within 12 months of application.	
	Affected area of ≥10% on HRCT.	
Progression	In the 2 years prior to the application, one of the following: Relative decline of FVC% predicted of ≥10% Relative decline of FVC% predicted of ≥5% and < 10% with either worsening respiratory symptoms or increased fibrosis on HRCT	Two or more of the following within the past 12 months without an alternative explanation: Worsening respiratory symptoms Physiological deterioration: Absolute decline in FVC% predicted ≥5%, or Absolute decline in DLCO %predicted ≥10% Radiological progression: Increased extent or severity of traction bronchiectasis/bronchiolectasis New ground glass opacity with traction bronchiectasis New fine reticulation Increased extent or increased coarseness of reticulation New or increased honeycombing Increased lobar volume loss

Abbreviations: ATS/ERS/JRS/ALAT, American Thoracic Society, the European Respiratory Society, the Japanese Respiratory Society, and the Latin American Thoracic Association; DLCO, diffusing capacity for carbon monoxide; FEV_1_, forced expiratory volume in 1 s; FVC, forced vital capacity; HRCT, high‐resolution computed tomography; IPF, idiopathic pulmonary fibrosis; PBS, pharmaceutical benefits scheme; PF‐ILD, progressive fibrosing interstitial lung disease; PPF, progressive pulmonary fibrosis.

Disease subgroups that have the potential to fulfil such criteria include idiopathic fibrotic non‐specific interstitial pneumonia (NSIP), fibrosing organizing pneumonia, fibrotic hypersensitivity pneumonitis (HP), fibrotic autoimmune‐associated ILD, fibrotic sarcoidosis, unclassifiable fibrotic ILD and fibrotic occupational lung diseases. It should be emphasized that the terms PPF and PF‐ILD do not denote a separate diagnostic entity, but rather define disease behaviour to guide therapeutic decisions. For consistency, ‘PPF’ will be used throughout the following sections.

#### Treatment approaches for PPF

##### First‐line therapeutic strategies and consideration of ‘treatment failure’

Implicit in the definition of PPF is progression of fibrosis *despite management considered optimal by individual clinicans*. There are important caveats to this construct in that (a) ‘first‐line’ therapy for many non‐IPF ILD subgroups is frequently consensus‐based with limited high‐quality evidence; and (b) patients with unclassifiable ILD may progress without having received any prior pharmacological therapy, due to uncertainty about the best treatment strategy. Immunosuppressive agents (including corticosteroids, mycophenolate mofetil, cyclophosphamide, azathioprine, methotrexate and rituximab, among others) are often used in non‐IPF ILD, with specific evidence and rationale beyond the scope of this document.^59^, ^60^, ^61^ As for all patients suffering from chronic respiratory disease, non‐pharmacological interventions (e.g., pulmonary rehabilitation, vaccinations, oxygen, transplant referral) should be considered for patients with PPF, providing complementary benefits alongside drug therapies.

**FIGURE 1:**
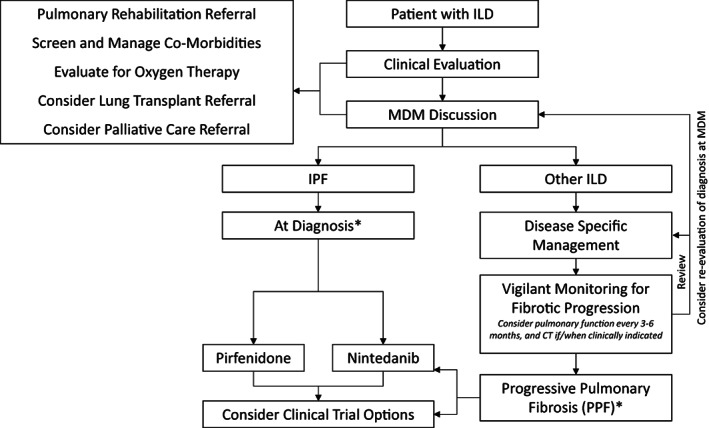
Suggested algorithm for the management of IPF and PPF. *Subsidized prescribing criteria will vary depending on jurisdiction. Observation of fibrotic disease progression in an ILD other than IPF should prompt review of the disease specific management approach, consideration of re‐evaluation of the diagnosis at an ILD‐MDM and consideration of nintedanib should it be accessible. Enrolment in clinical trials should be considered for all patients with IPF and PPF. Alongside pharmacologic therapies, all ILD patients should be considered for supportive therapies as well as lung transplantation and palliative care where relevant. ILD, interstitial lung disease; MDM, multi‐disciplinary meeting; PPF, progressive pulmonary fibrosis.

In the longitudinal assessment of a patient with fibrotic ILD, it is now increasingly important to regularly reassess disease severity clinically, and with pulmonary function and high resolution CT. Progression should always prompt diagnostic reassessment of the ILD, as the passage of time and observation of an individual's physiologic and radiologic disease trajectory, and response, or lack thereof to other therapies, may trigger disease re‐classification. This may be particularly relevant for fibrotic ILDs previously considered unclassifiable. Despite demonstrating progressive disease behaviour, certain ILDs may still be best treated with immunosuppression such as CTD‐ILD, or antigen avoidance in fibrotic hypersensitivity pneumonitis, potentially alongside the addition of nintedanib, as discussed below. A suggested pathway for the pharmacologic management of IPF and PPF is provided in Figure 1.

#### Evidence for nintedanib and pirfenidone in the treatment of PPF

The INBUILD study was a phase 3 randomized controlled trial recruiting 663 patients.^5^ Patients were required to have non‐IPF fibrosing lung disease affecting greater than 10% of lung volume on CT and evidence of progression despite management within the preceding 24 months. This study showed that nintedanib was beneficial, with patients on treatment declining in FVC by 80.8 mL/year compared with 187.8 mL/year in the placebo group, giving a difference of 107.0 mL (95% CI 65.4–148.5, *p* < 0.001). A subgroup analysis of this trial, whilst underpowered, showed that the effect was consistent across the different diagnostic subgroups including fibrotic HP, autoimmune ILD as well as idiopathic and unclassifiable ILDs.^62^ An open label extension of the long term safety of nintedanib in this population is currently underway (INBUILD‐ON, NCT03820726). Nintedanib received a conditional recommendation in favour for this indication in the updated ATS/ERS/JRS/ALAT guidelines and is now approved in Australia for use in patients with PPF.^2^, ^63^ Concomitant use of nintedanib and mycophenolate mofetil within the SENSCIS trial shows a manageable gastrointestinal side effect profile in scleroderma‐ILD patients, suggesting this may be tolerable in other disease groups.^64^

Pirfenidone, on the other hand, has not received local approval or guideline recommendations for use in PPF, based on the current evidence.^2^, ^65^, ^66^ In the unclassifiable ILD study, a phase 2 randomized controlled trial recruiting 253 patients with progressive fibrosing unclassifiable ILD, the primary efficacy outcome measured was a mean change in FVC from baseline over 24 weeks on daily home spirometry.^67^ While pirfenidone appeared to show efficacy with FVC falling by 87.7 mL in the treatment arm compared to 157.1 mL with placebo, home spirometric data provided excessive intra‐individual variability as well as physiologically implausible results. As such, pre‐specified statistical models were unable to be used for analysis of the primary endpoint. Key secondary endpoints including laboratory‐measured FVC suggested efficacy for pirfenidone (−17.8 mL vs. −113.0 mL; treatment difference 95.3 mL [95% CI 35.9–154.6], *p* = 0.002).

The unclassifiable ILD trial was followed by the RELIEF study, a phase 2b trial including patients with PPF due to autoimmune disease‐ILD, fibrotic HP, asbestosis or fibrotic NSIP.^68^ This study was prematurely terminated after only 127 patients had been randomized due to slow recruitment. Whilst underpowered, this study did suggest a slower decline in FVC predicted in the treatment arm versus placebo (−36.6 mL vs. −114.4 mL; treatment difference of 80 mL, 95% CI −40.0 to 210.0, *p* = 0.21). A meta‐analysis of the two studies showed a statistically significant decline in markers of disease progression with pirfenidone, however, overall treatment effect estimates were small, leading to uncertainty about the clinical impact of this therapy in PPF.^65^ Further evaluation of pirfenidone in non‐IPF patients with disease progression is needed.

- *PPF refers to non‐IPF ILD which is progressive despite treatment with optimal therapy*.
- *Disease progression in non‐IPF ILDs should prompt reassessment of diagnostic classification and consideration of the role of other disease modifying therapies*.
- *Nintedanib should be considered in patients with PPF, with evidence for treatment benefit across a range of ILD subgroups*.
- *Low quality evidence suggests a treatment benefit for pirfenidone in PPF*.

### Clinical trials for IPF and PPF

**FIGURE 2:**
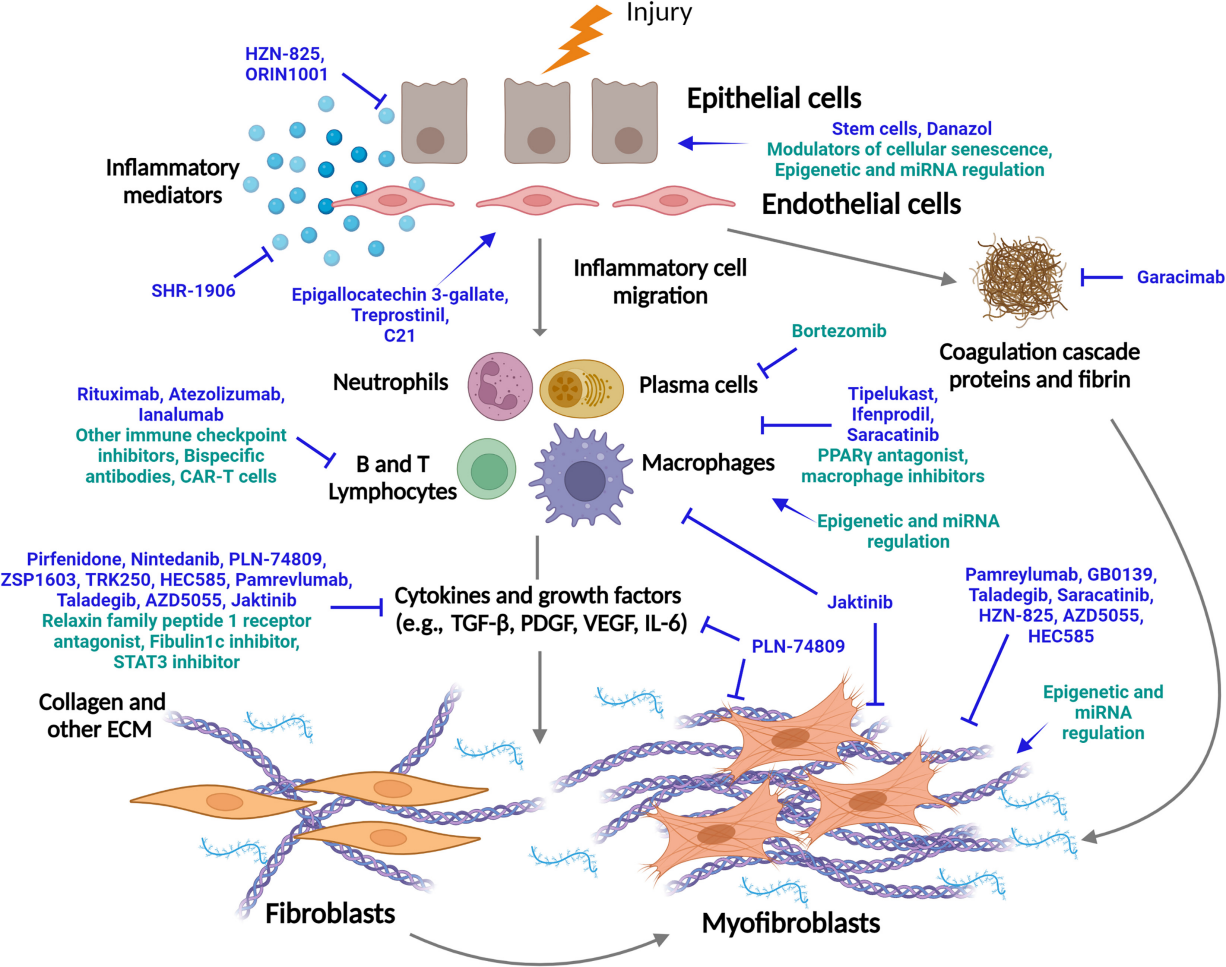
Pathogenic pathways of IPF and potential clinical trial targets. Current (blue) and proposed (green) molecules assessed in clinical trials for the treatment of idiopathic pulmonary fibrosis. Modulators (→) and inhibitors (┬) are shown directed at different components of the fibrosis pathway. Created with Biorender.com.

The advent of anti‐fibrotic therapy has been a major breakthrough in the management of IPF. The routine and now widespread use of nintedanib and pirfenidone is the result of multiple, robust, randomized clinical trials involving international collaboration. It is, however, important to acknowledge that patients with IPF on either anti‐fibrotic continue to progress and exacerbate and may die of their lung disease. Better treatments, likely in combination with current anti‐fibrotics, will be identified, through increasingly sophisticated clinical trials. Further unravelling of the mechanisms underlying the pathogenesis of IPF will enable more potential therapeutic targets to be identified (Figure 2). Novel drugs are being developed to modify a range of key cellular and metabolic pathways in IPF, ranging from inhibitors of proinflammatory and profibrotic molecules through to immunomodulatory molecules and inhibitors of cellular senescence. Some of the key pathways of particular interest that are being targeted for the development of novel drugs and likely to be in clinical trial over the next few years are outlined in Table 3 and Figure 2.

**TABLE 3:** Novel targets under investigation for future clinical trials.

Molecules or pathway	Mechanism of action	References
Enhancing bone morphogenetic protein receptor 2 (BMPR2) signalling	Restores pSmad1/5/8 signalling and inhibits TGFβ‐induced pSmad2 expression, reducing TGFβ signalling. Blocks fibroblast differentiation and collagen production	69, 70
Epigenetic and microRNA regulation	DNA methylation inhibitors and microRNA mimics and inhibitors restore DNA methylation and microRNA levels, respectively to normal levels	71, 72
Fibulin1c inhibitors	Blocks the interaction of fibulin1c with latent TGF‐β binding protein‐1 to prevent TGF‐β activation and signalling. Blocks fibroblast differentiation and collagen production	73, 74
Immune cell regulators (e.g., checkpoint inhibitors)	Checkpoint inhibitors regulate immune/host cell interaction to regulate immune response which inhibits T cell differentiation and in turn blocks fibroblast differentiation and migration and inhibits collagen production	75, 76
Inhibitors of profibrotic macrophages	Selective depletion of profibrotic macrophages which are one of the key cells that release mediators to stimulate fibroblast differentiation and collagen production	77, 78
Modulators of cellular senescence	Senescent‐selective apoptosis (senolytic) and senescence‐associated secretory phenotype suppression (senomorphic) to induce myofibroblast apoptosis and overcome senescence in epithelial cells, respectively	79
Peroxisome proliferator‐activated receptor‐γ (PPARγ) agonists (e.g., rosiglitazone, pioglitazone)	Inhibits inflammation, smooth muscle contraction and fibrosis. Reduces TGFβ1, IL‐4, ‐5, ‐6, ‐11 and ‐13 which are profibrotic mediators	70, 80
Plasmablast and plasma cell inhibitors (e.g., bortezomib)	Targets and eliminates plasmablasts and plasma cells in the serum and tissue	81
Relaxin family peptide 1 (RXFP1) receptor agonists (e.g., CGEN25009)	Inhibits TGFβ1/Smad2 signalling and upregulates MMP‐2 and 9. Inhibits and reverses fibrosis by preventing collagen production and increasing collagen degradation	70, 82, 83, 84
STAT3 inhibitors	Block pathways regulated by IL‐6 family cytokines that are associated with fibrosis. Blocking STAT3 has wide ranging effects on immune cells, epithelial cells and fibroblasts which ultimately blocks fibroblast differentiation and collagen production	84, 85, 86

Abbreviations: BMPR2, bone morphogenetic protein receptor type 2; DNA, deoxyribonucleic acid; IL, interleukin; MMP, matrix metalloproteinase; RNA, ribonucleic acid; STAT, signal transducer and activator of transcription; TGF, transforming growth factor.

Australian patients with IPF and their caregivers have highlighted the identification of medications that can reverse lung scarring and improve lung function as one of their top research priorities.^87^ The effective management of IPF symptoms was also high in their responses. There are currently numerous clinical trials in Australia and New Zealand addressing these priorities. It is important to recognize and appreciate the benevolence of many IPF patients to improve the lives of future IPF patients through their participation in IPF research. Reassuringly, most clinical trials allow background anti‐fibrotic therapy with the experimental agent being evaluated on top of standard of care. Additionally, clinical trials offer another therapeutic avenue for patients who are intolerant of current anti‐fibrotic therapies. This position paper recognizes the challenges to clinical trial participation for rural and remote patients and their treating clinicians. Many trials provide financial reimbursement for travel and accommodation. Efforts are being made to improve clinical trial access to this group of patients. A contemporaneous list of active ILD clinical trials in Australia and New Zealand is provided through Lung Foundation Australia's Pulmonary Fibrosis Australasian Clinical Trials Network (PACT) (https://pact.lungfoundation.com.au/).

- *All patients with IPF and PPF should be presented with the option to participate in clinical trials*.
- *A contemporaneous list of active ILD clinical trials and recruiting centres in Australia and New Zealand is provided through Lung Foundation Australia's Pulmonary Fibrosis Australasian Clinical Trials Network (PACT)* (https://pact.lungfoundation.com.au/).

### Acute exacerbations of IPF

Acute exacerbation of IPF (AE‐IPF) is defined as an acute, clinically significant respiratory deterioration in a patient with a previous or concurrent diagnosis of IPF, characterized by evidence of new, widespread alveolar abnormality not explained by cardiac failure/fluid overload (Table 4).^88^ In some cohorts, up to 10% of patients experience an acute exacerbation per annum, with associated in‐hospital mortality of over 50% and median survival post exacerbation of 3–4 months.^88^, ^89^ The prognosis of AE‐IPF is poor. Up to 46% of deaths in IPF are preceded by an acute exacerbation.^90^ When respiratory failure from AE‐IPF develops, it is associated with high in‐hospital mortality (>50% in most series).^91^, ^92^ Importantly, AE‐IPF may be the initial presentation for someone not previously known to have IPF.

**TABLE 4:** Criteria for acute exacerbation of IPF.^88^

Acute respiratory deterioration of typically <1 month duration in someone with IPF
Extra‐parenchymal cause excluded (e.g., pneumothorax, pleural effusion, pulmonary embolism)
New, bilateral ground glass opacity and/or consolidation on CT
Not explained by congestive cardiac failure of fluid overload

Abbreviations: CT, computed tomography; IPF, idiopathic pulmonary fibrosis.

Exacerbations may be of known (e.g., infection) or unknown aetiology. Although the precise pathogenesis of AE‐IPF remains uncertain, it appears likely that many exacerbations may be triggered by an external insult such as infection or micro‐aspiration,^91^ with little data to suggest any meaningful distinction between these two aetiologies. AE‐IPF are more common in patients with advanced lung disease, with low FVC being the most consistent risk factor.^90^, ^91^, ^92^ Other physiological parameters indicative of severe disease have also been associated with increased risk, including low DLCO, reduced 6‐minute walk distance (6MWD), pulmonary hypertension (PH) and poor baseline oxygenation. Other candidate risk factors for AE‐IPF include younger age, higher body mass index, co‐existing coronary artery disease, surgery (particularly thoracic surgery) and a history of prior AE‐ILD.^93^, ^94^ Despite prior concerns that bronchoscopy with bronchoalveolar lavage may precipitate AE‐IPF, there are increasing reassuring data to support its use.^95^

Preventive strategies include vaccination (e.g., influenza, pneumococcal, COVID‐19), and caution around surgery, particularly cardiothoracic surgery.^96^, ^97^ Regional anaesthesia is preferred over general anaesthesia, where possible. The indication for surgery should be balanced against the risk of acute exacerbation and its attendant mortality, and communicated to the patient during informed consent.

There remain no proven therapies for AE‐IPF. Management consists of supportive care with a focus on palliation of symptoms, supplemental oxygen to correct hypoxaemia and consideration of broad spectrum antibiotics and/or antiviral agents to cover possible infection. Non‐invasive ventilation and high flow oxygen are often initiated, but data to support these therapies are limited. The ATS/ERS guidelines recommend against mechanical ventilation, stating that ‘the majority of patients with respiratory failure due to IPF should not receive mechanical ventilation, but mechanical ventilation may be a reasonable intervention in a minority’ (weak recommendation, low quality evidence).^6^ The decision to undertake mechanical ventilation in this setting needs to be carefully considered, with the in‐hospital mortality being as high as 90% in this population.^88^ In individual situations (e.g., following identification of a specific treatable cause for the exacerbation such as infection or pulmonary embolism, or as a bridge to lung transplant), such a decision to use mechanical ventilation and/or extracorporeal membrane oxygenation (ECMO) may be considered appropriate.

Corticosteroids are often used in AE‐IPF, although there is no controlled clinical trial evidence to support this treatment approach. The majority (63%) of surveyed pulmonologists treat AE‐IPF with methylprednisolone or equivalent with a dose of 500–1000 mg for 3 days followed by a long taper, with another 11% using pulsed high‐dose steroids for 3 days only.^98^ The ATS/ERS guidelines include a weak recommendation that the majority of patients with AE‐IPF should be treated with corticosteroids.^6^ A recent small retrospective evaluation of corticosteroid treatment for AE‐IPF at a single centre, observed reduced survival in the group that received corticosteroids, and suggested that corticosteroids might actually contribute to adverse outcomes for AE‐IPF.^99^ However, in this non‐randomized study, the group that received corticosteroids had more severe disease at baseline and worse vital status at presentation. A recent, small, retrospective study reported that nintedanib commenced during AE‐IPF was associated with a lower 90‐day mortality, however, firm conclusions are not possible with this uncontrolled data.^100^ AE‐IPF unfortunately remains a challenging clinical scenario, with limited evidence to guide management.

Observational cohort studies of a number of other therapies have been conducted. However, a recent randomized controlled trial showed that adding intravenous cyclophosphamide to standardized high dose glucocorticoids increased 3 month mortality, providing evidence against the use of cyclophosphamide in this setting. There are no randomized controlled trials for the other agents, and the routine use of these outside a clinical trial setting is not supported by the current evidence.^101^

Emergent lung transplantation is rarely performed, and where possible avoided through timely elective transplant evaluation and waitlisting. However, patients may deteriorate unexpectedly, in particular patients with IPF. In Australia and New Zealand, there are provisions in place to facilitate access to donor lungs in urgent scenarios. AE‐IPF is not a contraindication to transplantation, but transplantation in the rapidly deteriorating patient is associated with worse outcomes. In one study of 37 IPF patients waitlisted for transplant who suffered an AE‐IPF, 28 survived to transplantation. Eleven patients were placed on ECMO support with only four surviving to transplant in that setting.^102^ One and three year survival for these 28 transplanted patients (71% and 60%, respectively) was dramatically lower than for patients transplanted with ‘stable’ IPF (94% and 90%, respectively). Transplantation is seldom offered in this scenario, but is considered in highly selected cases. These data emphasize the importance of preparing patients early for transplantation with consideration of early referral to a transplant centre.

- *The prognosis of AE‐IPF is poor. Available treatment options are limited, and not supported by controlled trial data*.
- *General anaesthesia and cardiothoracic surgery have been associated with increased risk for AE‐IPF. This should be considered in the pre‐operative evaluation and informed consent of IPF patients*.
- *Corticosteroids, typically in high doses, are frequently utilized in the management of AE‐IPF, but data on efficacy are lacking*.
- *Anti‐fibrotic therapies have no proven role for initiation in the acute setting*.
- *Outcomes of lung transplantation during AE‐IPF are poor, but might be appropriate in highly selected cases*.

### Treatment and impact of co‐morbidities

As IPF is a disease seen with advancing age, comorbid conditions are common at time of diagnosis and throughout the disease course.^103^ Individual comorbidities such as PH, heart disease and lung cancer are associated with increased mortality in IPF cohorts.^104^, ^105^, ^106^ The overall number of comorbidities is also an important predictor of survival, with one recent study showing that a greater number of comorbidities was associated with a higher risk of death within each GAP stage of IPF disease severity.^103^, ^107^ Some conditions including lung cancer, chronic obstructive pulmonary disease (COPD)/emphysema and coronary artery disease are highly prevalent in patients with IPF due to the shared risk of tobacco exposure. Others such as obstructive sleep apnoea (OSA) causing repetitive nocturnal hypoxaemia may be important contributors to the development and/or progression of fibrotic lung disease.^108^, ^109^, ^110^ Targeted treatment of conditions may be considered, even where specific evidence may be lacking, for the indication of improving IPF‐specific outcomes and quality of life.

#### Pulmonary hypertension

PH relating to IPF (and other ILD) generally falls within group 3 of the World Health Organization (WHO) classification scheme, (precapillary PH associated with lung diseases and/or hypoxia). Effective pharmacological treatment for this complication of advancing fibrotic lung disease has been elusive, with most studies failing to show any clinical benefit for vasodilator therapy in ILD patients (Table 5).^114^ Furthermore, some therapies, (i.e., ambrisentan and riociguat), have been associated with increased ILD progression and respiratory hospitalization.^112^, ^115^ Consideration of PH specific therapies in people with IPF/PPF should only be considered through expert PH centres.^117^

**TABLE 5:** Recent seminal studies with vasodilator therapies in IPF and other ILD cohorts.

Study	Year	Population	Number patients	Primary endpoint	Outcome
**INCREASE** ^111^ Treprostinil Versus placebo *Phase 2/3*	2021	ILD with PH confirmed by right heart catheter	326	Change in 6MWD at 16‐weeks	Improved 6MWD in treatment arm of 31.12 m (95% CI, 16.85–45.39; *p* < 0.001); improved NT‐proBNP, reduced clinical worsening, compared with control arm.
Sildenafil plus pirfenidone versus placebo plus pirfenidone^68^ *Phase 2b*	2021	IPF with DLCO ≤40% predicted and mPAP ≥20 mm Hg	177	Proportion with disease progression (change in 6MWD, respiratory hospitalization, death) at 52‐weeks	No difference in the primary endpoint, between‐group difference 3.06% (95% CI –11.30 to 17.97; *p* = 0.65).
**RISE‐IIP** ^112^ Riociguat versus placebo *Phase 2b*	2019	IIP with PH confirmed by right heart catheter	147	Change in 6MWD at 26‐weeks	No difference in the primary endpoint or time to clinical worsening; trial terminated early due to increased SAEs including death in treatment arm.
**INSTAGE** ^113^ Sildenafil plus nintedanib versus placebo plus nintedanib *Phase 3*	2018	IPF patients with DLCO ≤35% predicted	274	Change in baseline total SGRQ score at 12‐weeks	No difference in mean change in SGRQ score (treatment arm −1.28 points, control arm −0.77 points; *p* = 0.72). No difference in dyspnoea scores or safety.
**BPHIT** ^114^ Bosentan versus placebo *Phase 2*	2014	Fibrotic IIP with right heart catheter confirmed PH	60	Fall from baseline pulmonary vascular resistance index of 20% or more at 16 weeks	No difference in invasive pulmonary haemodynamics, functional capacity or symptoms.
**ARTEMIS‐IPF** ^115^ Ambrisentan versus placebo *Phase 3*	2013	IPF patients with ≤5% honeycombing on HRCT scan	492	Time to disease progression (death, respiratory hospitalization, decrease in FVC and DLCO); 48‐week assessment	Increased disease progression in treatment arm (90 [27.4%] vs. 28 [17.2%] patients; *p* < 0.010; hazard ratio, 1.74 [95% CI, 1.14–2.66])
**STEP‐IPF** ^116^ Sildenafil versus placebo *Phase 3*	2010	IPF patients with DLCO ≤35% predicted	180	Proportion of patients with an increase in 6MWD ≥20%	No difference in proportion meeting primary endpoint (treatment arm 10%, control arm 7%; *p* = 0.39); some secondary endpoints improved with treatment

Abbreviations: 6MWD, six‐minute walk distance; DLCO, diffusing capacity for carbon monoxide; FVC, forced vital capacity.; HRCT, high resolution computed tomography; IIP, idiopathic interstitial pneumonia; ILD, interstitial lung disease; IPF, idiopathic pulmonary fibrosis; mPAP, mean pulmonary artery pressure; NT‐proBNP, N‐terminal pro‐B‐type natriuretic peptide; PH, pulmonary hypertension; SAE, serious adverse event; SGRQ, St George's Respiratory Questionnaire.

While not designed as a treatment of PH in ILD trial, STEP‐IPF promisingly demonstrated a potential signal for improvement in gas transfer, oxygenation, dyspnoea and quality of life with sildenafil in IPF patients with advanced disease (DLCO <35%), even though the study's primary endpoint of change in six‐minute walk distance (6MWD) was not met.^116^ Subsequently, sildenafil combined with anti‐fibrotic treatment has been investigated. A multicentre international randomized, double blinded study evaluated pirfenidone plus sildenafil versus pirfenidone plus placebo in IPF patients with advanced disease (DLCO ≤40% predicted) and mean pulmonary artery pressure ≥ 20 mm Hg.^68^ At 52 weeks, there was no difference between the two groups in the primary endpoint of disease progression. Also not designed as treatment of PF in ILD trial, the INSTAGE study evaluated sildenafil versus placebo with background nintedanib in advanced IPF (DLCO ≤35% predicted), and demonstrated no change in the St George Respiratory Questionnaire primary endpoint, nor other indices of dyspnoea.^113^ A prespecified subgroup analysis in those with echocardiographic signs of right heart dysfunction at baseline did not differ from the primary study findings.^118^ Neither study of sildenafil with anti‐fibrotic therapy showed any concerning safety signals. Very recently, a retrospective observational cohort study suggested a potential survival benefit with sildenafil in ILD‐PH patients, mostly IPF, where PH had been confirmed by invasive right heart catheterisation.^119^

In contrast to previous studies, inhaled treprostinil has recently been shown in an early phase study to improve 6MWD by 31 m as well as reduce the risk of clinical worsening (defined as cardiopulmonary hospitalization, >15% reduction in baseline 6MWD, death or transplant) (HR 0.61; 95%CI 0.40–0.92, *p* = 0.04) in an ILD cohort with confirmed pre‐capillary PH.^111^ Additionally, N‐terminal pro‐B‐type natriuretic peptide (NT‐proBNP) was observed to decrease by 15% from baseline with inhaled treprostinil and increase by 46% in the control arm at week 16. There were also fewer exacerbations of the underlying lung disease in the treatment group. No serious safety signals were seen. A larger phase 3 clinical trial in IPF is currently underway to evaluate the impact on FVC decline, although is not specifically evaluating its role in IPF‐PH.

- *Ambrisentan, bosentan and riociguat are contraindicated in PH associated with IPF*.
- *Sildenafil might be considered on a case‐by‐case basis, through a PH‐expert centre, in IPF patients with confirmed precapillary PH where access to specific IPF‐PH clinical trials are not possible*.
- *Inhaled treprostinil may improve exercise capacity and attenuate clinical worsening in ILD patients with confirmed pre‐capillary PH*.

#### Obstructive sleep apnoea

Higher rates of OSA have been observed in IPF and ILD cohorts relative to the general population, with a recent meta‐analysis estimating an overall prevalence of 61% in patients with ILD, (32% classified as mild, 17% moderate, and 9% severe).^120^ This association may relate to alveolar micro‐injury secondary to traction in the lung peripheries, as a consequence of repetitive exaggerated changes in pleural pressure during apnoea.^121^ OSA has been suggested as an independent risk factor for developing ILD in the Multi‐Ethnic Study of Atherosclerosis (MESA) study.^122^ An apnoea hypopnoea index (AHI) >15 (indicating moderate to severe OSA), was associated with a 35% increased odds of interstitial lung abnormalities (ILA) on CT imaging (95% CI, 13–61%; *p* = 0.001). This association was strongest in those with BMI <25 kg/m^2^. Serum markers of alveolar epithelial injury and extra‐cellular matrix remodelling were also associated with OSA severity in this community‐based cohort.

Recently, the ‘hypoxic burden’ index (the area under the desaturation curve associated with respiratory events) during sleep has been identified as a robust predictor of cardiovascular mortality in general OSA populations.^123^ A number of studies have confirmed the high degree of sleep‐related hypoxaemia in IPF/ILD patients, and furthermore, have shown associations between nocturnal hypoxaemia and the development of PH and increased mortality in these cohorts.^110^, ^124^, ^125^ Nocturnal oxygen desaturation occurs as a direct consequence of OSA but may also relate to other physiologic derangements of ventilation and gas exchange in patients with IPF/ILD.^126^

Whilst very little high‐quality IPF/ILD‐specific evidence is available, general OSA interventional studies provide a framework for initiating therapy. It is reasonable for continuous positive airway pressure (CPAP) to be considered for patients with moderate to severe OSA, particularly in those with daytime somnolence. Specific issues for patients with fibrotic lung disease (such as excessive cough, mood disturbance and reduced lung compliance) need to be considered and may impact tolerability. Limited data in small uncontrolled cohorts suggest improved quality of life and possible mortality benefits in IPF‐OSA patients using CPAP.^127^, ^128^, ^129^ However, a recent retrospective study of 131 IPF patients with OSA, reported that neither severity of sleep disordered breathing or compliance with CPAP were associated with improved mortality or progression‐free survival.^130^

Hypopnoea is more common than apnoeas in patients with IPF/ILD, with the hypothesis that some are more vulnerable to sleep‐related ventilatory control instability (enhanced loop gain) due to chronic hypoxaemia.^131^ Patients exhibiting the enhanced loop gain endotype (rather than predominantly upper airway collapse endotype) may respond to oxygen supplementation during sleep rather than CPAP, however, further research is needed.^132^

- *OSA and nocturnal hypoxaemia occur frequently in patients with IPF and other fibrotic lung diseases. Clinicians may consider offering CPAP and/or nocturnal oxygen supplementation in this setting*.

#### Combined pulmonary fibrosis with emphysema (CPFE)

Combined pulmonary fibrosis with emphysema (CPFE) is a term coined to characterize the overlapping lung pathologies of ILD and emphysemaobserved in a subset of IPF and other ILD patients, often in the context of previous or current heavy tobacco exposure.^133^ A recent ATS/ERS/JRS/ALAT Research Statement has been published on this entity.^134^ Patients with CPFE are at higher risk of complications such as PH, lung cancer and progression to hypoxaemic respiratory failure, than those with pulmonary fibrosis alone.^135^, ^136^ Those with IPF as their underlying ILD follow a worse disease trajectory than other subtypes associated with the syndrome.^137^

Smoking cessation is an important intervention for current smokers. Where there is demonstrable airflow obstruction or suggestive symptoms of COPD, guideline‐directed inhaler therapy may lead to substantial benefit.^138^, ^139^ Pulmonary rehabilitation is an important strategy for patients with CPFE, as it is for patients with either condition on its own, and is discussed in further detail below. Supplemental oxygen may help alleviate pulmonary hypoxic vasoconstriction as a contributing factor to PH in CPFE as detailed below.

Acute exacerbations are another important cause of morbidity and mortality in CPFE, and can be of COPD‐type or IPF‐type, according to published definitions.^88^, ^138^, ^140^ Treatment for COPD‐type exacerbations may incorporate bronchodilators, systemic corticosteroids, anti‐microbials, oxygen and non‐invasive ventilatory support, depending on the severity.^138^ Patients experiencing IPF‐type exacerbations with acute hypoxic respiratory failure and diffuse ground glass opacification on HRCT may have poorer outcomes.^140^ Management is discussed in greater detail earlier (Section 3.5) in this document.

Little data exist specifically for the use of anti‐fibrotic therapy in CPFE patients. The ASCEND trial in pirfenidone excluded patients with FEV1/FVC ratios <0.8 after earlier studies suggested lack of efficacy in this sub‐group.^7^, ^141^ The INPULSIS studies with nintedanib included IPF patients with concomitant radiologic emphysema, with treatment shown to be as effective in slowing the decline in FVC in these subjects as in those without emphysema.^24^, ^142^ Decline in FVC is of limited prognostic value in CPFE, and a preserved or stable FVC may be falsely reassuring, however, changes in FEV1 or DLCO are emerging as more robust predictors of survival in CPFE case series.^143^, ^144^

- *CPFE has emerged as a distinct clinical syndrome with increased risks of PH, lung cancer and hypoxic respiratory failure*.
- *Holistic treatment should include non‐pharmacological therapies and treatment of airways disease with inhaled bronchodilators and corticosteroids*.
- *Treatment of the underlying fibrosis in CPFE will depend on the ILD subtype. In those with IPF or PPF, anti‐fibrotic therapy may be considered*.

#### Lung cancer

IPF is considered an independent risk factor for lung cancer even after adjusting for age, gender and tobacco exposure.^145^ Recent genomic profiling in IPF and non‐small cell lung cancers (NSCLC) reveal overlapping upregulated gene expression in collagen organization, matrix adhesion and cell cycle control pathways.^146^, ^147^ The incidence of lung cancer in IPF patients is difficult to gauge in the absence of routine screening, but is estimated to be five‐fold higher than the general population, with greatest risk in males with a history of smoking.^145^, ^148^, ^149^ This association has prompted almost half of 494 physicians involved in an international survey to perform regular low dose HRCT screening for lung cancer in patients with IPF.^150^ A recent multi‐centre European study of 3178 patients with IPF identified 324 lung cancers (10.2%), the presence of which was not surprisingly associated with worse all‐cause mortality.^151^ Interestingly, decreased mortality occurred with anti‐fibrotic treatment in patients with IPF and lung cancer, supporting continuation of anti‐fibrotic treatment despite the development of lung cancer.^151^ A lower incidence of AE‐IPF has been observed with perioperative pirfenidone in people with IPF undergoing lung cancer resection.^152^, ^153^ Patients with CPFE have a higher lung cancer risk than patients with IPF or emphysema alone.^154^ Squamous cell carcinoma followed by adenocarcinoma are the most common histopathological subtypes in IPF and CPFE cohorts.^148^, ^151^, ^155^

Surgical resection is an option for patients with early‐stage cancer and sufficient pulmonary reserve. Surgically treated patients had improved all‐cause mortality compared to those with technically operable lung cancer who did not undergo surgery.^151^ Sub‐lobar resection may be better tolerated than lobectomy, with some data to suggest a lower risk of acute exacerbation and in‐hospital complications in IPF patients.^156^ A recently published large multi‐centre randomized controlled trial in early stage NSCLC demonstrated superiority for overall survival and non‐inferiority for relapse‐free survival with segmentectomy compared with the gold standard lobectomy.^157^ Although this study excluded patients with pulmonary fibrosis, the findings are reassuring for a surgically conservative approach. A multi‐centre study addressing this question in IPF patients is currently underway.^158^ However, even when the lung cancer is surgically resectable, patients with concomitant pulmonary fibrosis do worse than those without. In a study of over 2000 patients undergoing surgical resection of NSCLC, those with ILD had significantly poorer overall and cancer‐specific survival than those without ILD (overall survival: 40.4% vs. 72.0%, *p* < 0.01; cancer‐specific survival 55.4% vs. 78.6%, *p* < 0.01).^159^

Chemotherapy, tyrosine kinase inhibitors and immunotherapy may be considered in a minority of patients with IPF/ ILD and lung cancer. Cancer stage and molecular profile, performance status and severity of lung disease are important treatment modifiers. As with surgical cohorts, IPF patients have worse outcomes with these interventions compared with non‐IPF subjects. Risk of pulmonary toxicity manifesting as acute exacerbation is substantially increased with all treatment modalities and IPF/ILD is considered a relative contra‐indication for these options in some centres.^160^, ^161^ Stereotactic ablative radiotherapy carries a heightened risk of severe radiation pneumonitis in patients with ILD and in many centres ILD is considered a contraindication to this modality.^162^ Palliative measures may be the most appropriate care model for many IPF patients with lung cancer.

Nintedanib has some action against lung adenocarcinoma, with one study showing improved progression‐free survival when used in combination with docetaxel in general lung cancer patients with recurrence after first‐line treatment for advanced disease.^163^ A recently published randomized phase 3 study evaluated exacerbation‐free survival for nintedanib versus placebo in combination with carboplatin and nab‐paclitaxel in 243 IPF patients with advanced lung cancer.^164^ Whilst the primary endpoint of reducing the incidence of acute exacerbations was not met, the overall survival of IPF patients with non‐squamous cell cancer subtype was improved (HR 0.61; 95% CI 0.40–0.93).

- *Lung cancer is common in IPF. Some clinicians consider regular CT screening to identify lung cancers in IPF patients*.
- *Surgery is the first‐line option for appropriate patients, with recent evidence to support sub‐lobar resection as the optimal approach*.
- *Many of the usual treatment modalities for lung cancer management are limited in IPF due to high risk of pulmonary toxicity*.

## NON‐PHARMACOLOGICAL THERAPY

### Oxygen therapy

#### Continuous domiciliary and nocturnal oxygen

Since the previous position statement, new ATS guidelines for oxygen therapy have provided recommendations specific to patients with ILD.^1^, ^165^, ^166^ Domiciliary oxygen is indicated for IPF and other fibrotic ILD patients with resting partial pressure of arterial oxygen <55 mm Hg (or <60 mm Hg where there is evidence of PH). Evidence for these thresholds derives from historic randomized controlled studies conducted in COPD patients with very few data specific to ILD.^167^, ^168^, ^169^

Nocturnal hypoxaemia, even in the absence of OSA, is a common observation in ILD patients, with multiple studies consistently linking this phenomenon with poorer outcomes.^110^, ^126^ To date, very little data have been published on the impact of nocturnal oxygen supplementation in ILD patients. Nocturnal oxygen may be considered for patients with SpO_2_ <88% for >30% of sleep time, as for patients with other chronic respiratory diseases.^165^ Recent evidence suggests a benefit for nocturnal oxygen (versus CPAP) in ILD patients with hypopnoea‐predominant sleep disordered breathing, as detailed earlier (Section 3.6.2).^132^ This indication, however, is not endorsed in current oxygen guidelines and requires further investigation.^165^

#### Ambulatory oxygen

The AmbOx Study, published since the previous position statement, evaluated the impact of ambulatory oxygen in 84 ILD patients with isolated exertional hypoxaemia, defined as oxygen saturation ≤88% during 6‐minute walk test.^170^ The randomized open‐label crossover study demonstrated a potential improvement in health‐related quality of life (HRQOL) for patients using ambulatory oxygen over 2 weeks. Significant improvements were seen in total King's Brief Interstitial Lung Disease questionnaire (K‐BILD) scores (mean 55.5 [SD 13.8] on oxygen versus 51.8 [13.6] on no oxygen). There were also significant improvements in University of California, San Diego Shortness of Breath Questionnaire (UCSDSOBQ) and St George's Respiratory Questionnaire (SGRQ) scores. Breathlessness and activity, and chest symptom subdomains of each HRQOL scale were most markedly improved with the intervention. Psychological scores were not altered. The minimal important difference (MID) was not exceeded for the K‐BILD scores with ambulatory oxygen, although it was noted that the study population had more severe disease than the cohort upon which MID was calculated.^171^ The MID for UCSDSOBQ was exceeded with oxygen therapy in this study. Eagerly awaited are the results of the Australian‐led randomized, placebo‐controlled trial of ambulatory oxygen versus medical air in pulmonary fibrosis (PFOX; Pulmonary Fibrosis ambulatory Oxygen trial).^172^

Australian and New Zealand oxygen guidelines published in 2016 include the consensus‐based recommendation of ambulatory oxygen for those with isolated exertional desaturation, where a benefit in exercise capacity or dyspnoea can be demonstrated during a blinded trial of oxygen versus air.^165^ A TSANZ working group is currently reappraising the evidence to update these guidelines. The 2020 ATS guidelines recommend the prescription of ambulatory oxygen to adults with ILD who have severe exertional room air hypoxaemia.^166^

- *Continuous domiciliary or nocturnal oxygen may provide symptomatic benefit for ILD patients with resting or nocturnal hypoxaemia, although high‐quality evidence is lacking in this population*.
- *Ambulatory oxygen may be considered in a subset of ILD patients with exertional desaturation on the basis of improved activity and health‐related quality of life*.

### Pulmonary rehabilitation

Pulmonary rehabilitation is an effective therapy to reduce symptoms, enhance exercise capacity and improve health‐related quality of life in people with ILD. A Cochrane review^173^ including eight randomized controlled trials of pulmonary rehabilitation compared to usual care reported clinically important improvements in 6‐minute walk distance (mean 37 m, 95% CI 26–48 m) and dyspnoea (effect size −0.41, 95% CI −0.74 to −0.09) immediately following the program. Improvements in symptoms persisted at 6–12 months following completion of the program. Sustained improvements were more likely in those with higher FVC and less severe or no PH.^174^ Referral to pulmonary rehabilitation early in the disease course is therefore encouraged. The Australian and New Zealand Pulmonary Rehabilitation guidelines^175^ recommend that people with IPF undergo pulmonary rehabilitation, preferably in a program where supplemental oxygen can be delivered to ameliorate exercise‐induced desaturation. A referral to repeat pulmonary rehabilitation should be considered if functional capacity or symptoms worsen.^176^

Exercise training is a core component of pulmonary rehabilitation, including endurance training (typically walking or cycling) and strength training. Other components of pulmonary rehabilitation vary across programs but may include nutritional advice, stress management, occupational therapy, physiotherapy and education.^173^ Core educational topics that should be delivered during pulmonary rehabilitation in ILD have been identified^177^ including self‐management (importance of vaccination, regular exercise and good nutrition), keeping fit and strong after pulmonary rehabilitation, using oxygen therapy, managing symptoms (breathlessness, cough and fatigue) and managing mood. In Australia and New Zealand, most pulmonary rehabilitation programs will accept referrals for patients with ILD, and clinicians will individually tailor the program components according to their needs.

People with ILD experience barriers to attending pulmonary rehabilitation including a lack of perceived benefit, fear of breathlessness during exercise, the burden of travel to a pulmonary rehabilitation centre, inconvenient scheduling of sessions (particularly for those who are working) or caring responsibilities.^178^ Patients report that physician enthusiasm for pulmonary rehabilitation at the time of referral is a powerful facilitator of uptake^179^ and may overcome barriers related to patient knowledge and confidence. Barriers related to travel and transport may be addressed by remotely delivered models of pulmonary rehabilitation that can be delivered directly into the home, which are becoming more widely available in Australia and New Zealand. Remotely delivered pulmonary rehabilitation has been reported as safe, with outcomes that are similar to those delivered by centre‐based pulmonary rehabilitation programs.^180^ However, clinical trials of remote pulmonary rehabilitation have typically included patients with other chronic lung diseases, with only a small number of participants who had ILD. Special considerations for people with ILD undergoing remote pulmonary rehabilitation programs may include the capacity for monitoring of oxygen saturation and delivery of oxygen therapy during home‐based exercise training.

- *Pulmonary rehabilitation confers meaningful benefits in exercise capacity, symptoms and health‐related quality of life*.
- *Early referral for pulmonary rehabilitation is strongly encouraged for all patients with ILD, and consideration should be given to repeating pulmonary rehabilitation if symptoms progress*.

### General health measures and patient self‐management

Optimal management of ILD includes ensuring that patients and caregivers are actively engaged in care, with a sound understanding of their treatment plan and the importance of healthy behaviours. This is consistent with patient expectations of care in the anti‐fibrotic era, which includes working in partnership with health professionals to maintain good health and wellbeing.^181^ Patients express a strong desire for information on how to stay well with ILD and consider self‐management to be critically important.^177^, ^182^ General health measures and opportunities for self‐management should be discussed with all patients with ILD and their families, starting at the time of diagnosis. Important topics may include vaccination, avoiding infections, recognizing deterioration and seeking help, good nutrition, regular physical activity, medical considerations for planned travel, smoking and vaping cessation, managing mood, accessing social support and end of life planning.^183^

Whilst evidence for the impact of general health measures may be lacking in ILD, these interventions have implications beyond IPF disease. For example, maintaining good nutrition and a healthy body weight is often a consideration for transplant eligibility. Smoking cessation has critical implications for both transplant listing and use of oxygen therapy. Smoking has also been shown to decrease systemic exposure to both nintedanib and pirfenidone, and smoking cessation should be strongly advised when initiating these medications.^184^ The comprehensive management of patients with ILD includes the management of other co‐morbidities and disease‐related symptoms, for which many general measures will be highly relevant. For instance, depression and anxiety are common comorbidities in patients with ILD and are associated with higher symptom burden.^185^ There are no controlled studies addressing the management of anxiety and depression in IPF, but recognizing and treating these conditions is likely to be important to maximize wellbeing.

The management of patients with ILD involves many health professionals including their general practitioner, respiratory physician, ILD nurses, pharmacists, physiotherapists and other allied health professionals, as well as palliative care and transplant teams. Other medical teams may be involved with management of comorbid conditions. Patients with ILD identify coordination of care between health professionals as a high priority.^181^ As such, the patient's local general practitioner plays a vital role and should be involved at all stages.

- *Patients should be encouraged to engage in active self‐management starting from the time of diagnosis*.
- *It is important to provide patients with resources to support self‐management, such as those provided by Lung Foundation Australia* (https://lungfoundation.com.au/patients-carers/living-with-a-lung-disease/pf/overview/).

### Vaccination

Data specifically for the efficacy of vaccination in IPF are limited. However, it is appropriate to extrapolate much of the literature from the general population and studies in other chronic respiratory diseases. Patients with PPF receiving immunomodulation represent a unique cohort with specific vaccination requirements and contraindications. Vulnerable populations and First Nations people also have specific vaccination requirements. Country specific guidelines should be consulted for the latest recommendations (e.g., Australian Immunization Handbook: https://immunisationhandbook.health.gov.au/ or the New Zealand National Immunization Schedule: https://www.immune.org.nz). Annual influenza vaccination is strongly recommended for all adults with IPF and PPF, with the choice of vaccine determined by their age. Patients 70 years or older should undergo pneumococcal vaccination and it should also be strongly considered in ILD patients under the age of 70 years. Other vaccinations may be necessary during work‐up for transplantation.

- *National immunization guidelines should be followed*.
- *Patients with IPF and PPF are at higher risk of adverse consequences of respiratory virus infection and are therefore recommended for vaccination where available*.
- *Immunocompromised status presents an additional risk on top of the ILD, and should be considered in terms of eligibility and timing of vaccination, noting that live vaccines may be contraindicated depending on the level of immunocompromise*.

### COVID‐19

Patients with ILD are at increased risk of adverse outcomes from SARS‐CoV2 infection.^186^, ^187^, ^188^, ^189^, ^190^ Clinicians managing patients with ILD should remain up to date with recommendations regarding COVID‐19 vaccination for vulnerable groups. Notably, response to vaccination may be variably impaired by immunomodulation. Such patients may also benefit from other COVID‐19 pre‐exposure therapeutics if available.^191^

There are no specific data for the pharmacologic management of ILD patients infected with SARS‐CoV2. ILD patients with COVID‐19 should be considered for antiviral agents early after onset of symptoms (within 5 days). Grading the severity of COVID‐19 may be challenging in the context of baseline hypoxia. It is important to consider drug interactions with these antiviral agents. The University of Liverpool provides a useful online resource through which to check drug interactions with various COVID‐19 therapeutics (https://www.covid19-druginteractions.org/). Clinicians should remain current with other sources of COVID‐19 guidance.

- *ILD patients are at higher risk of adverse consequences of COVID‐19 and should follow guidelines for vaccination and if infected, have their eligibility for COVID‐19 therapeutics considered*.

### ILD specialist nurses

The role of specialist ILD nurses in the care of the ILD patient has been well‐described and evaluated.^181^, ^192^, ^193^, ^194^, ^195^, ^196^ Current international guidelines recommend that patients with IPF and their carers should have access to a specialist ILD nurse throughout the disease journey.^197^

Specialist ILD nurses have a key role to play in the management of patients with IPF including disease‐specific education, support of medication usage and management of their adverse effects, providing care coordination to manage the impact of disease progression and comorbid conditions and to providing support and advocacy throughout their disease course, from diagnosis to the palliative phase.

Challenges to implementing this recommendation include competing demands in healthcare funding, disparity in resource allocation in rural and regional areas compared to urban tertiary centres and institutional recognition of the value of this role. Lung cancer specialist nurses represent the most recognizably similar role in respiratory medicine suitable for benchmarking. They are present in 46% of centres providing the majority of lung cancer care in Australia,^198^ positively impact patient experience^199^ and are associated with better outcomes for people living with lung cancer.^200^ Additionally, recent Commonwealth of Australia funding of these positions represents a significant endorsement of their contribution.

- *Patients with IPF/PPF and their carers should have access to a specialist ILD nurse throughout their disease course*.

### Lung transplantation

**FIGURE 3:**
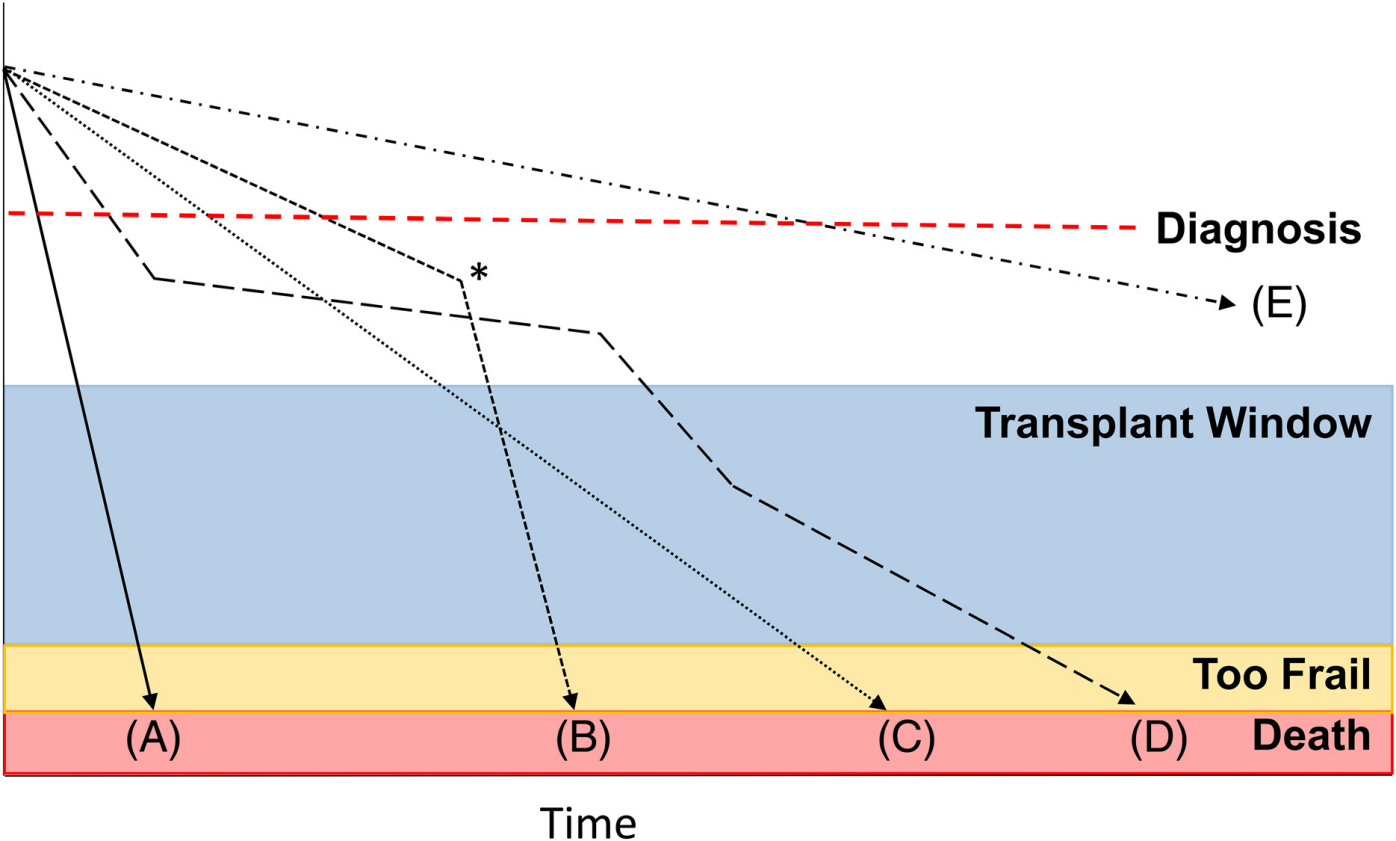
Transplant window and the unpredictable natural history of IPF necessitating referral early in the disease course. Given the unpredictable nature of IPF, transplant referral should be strongly considered at the time of diagnosis. (A) Rapidly deteriorating patient, whose diagnosis and transplant window occurs synchronously with a subsequent very narrow transplant window. (B) *Acute‐exacerbation of IPF prompting rapid deterioration. Outcomes of transplantation in this context are worse. (C) Linear progression, enabling easier assessment of when transplant window opens. (D) Step‐wise deterioration, a period of stability may give a false sense of reassurance. (E) Slowly progressive disease that may never come to transplant. Figure adapted from Ley et al.^203^

Despite recent advances in IPF therapy, there is still no cure for this relentlessly progressive disease. IPF is now the most common indication for lung transplantation globally.^201^ Referral for lung transplantation should occur early in the course of the disease since patients with IPF and PPF can face a protracted waitlist time, as the small chest cavity can limit the availability of suitable donor organs. As a result of the progressive nature of the disease, and reduced availability of suitable donors, mortality on the lung transplant waiting list is higher for IPF than for other diagnoses.^202^ A recent consensus document for the selection of lung transplant candidates recommends referral of appropriate candidates with a diagnosis of IPF at the time the diagnosis is made, regardless of the severity of their disease. This recommendation reflects the phenotypic heterogeneity of IPF, the difficulty in predicting disease course and overall poor prognosis of IPF patients compared with those with other indications for lung transplantation (Figure 3). For non‐IPF ILD, observation of progression over a 6‐month interval despite treatment has also been recommended as an indication for transplant referral.^204^ Early referral enables IPF and PPF patients to prepare themselves for future transplantation, and to address any issues that might preclude/delay transplantation (e.g., poor muscle strength, obesity and substance use).

Indications for active listing for lung transplantation are individualized, but reflect disease trajectory, anticipated prognosis, the development of PH and a person's anticipated wait list duration. An additional consideration for the timing of listing includes the possible presence of immune sensitisation, through the acquisition of antibodies targeting human leukocyte antigens (HLAs), which might narrow a candidate's donor pool. This is particularly common in multiparous women.

The presence of possible contraindications to transplantation should not necessarily preclude discussion with or referral to a transplant centre. The decision not to proceed to transplantation is best left to clinician teams with expertise in transplantation. Of particular note, age above 65 years is no longer an absolute contraindication to lung transplantation. Rather than chronological age alone, the physiological reserve of the patient as assessed by a variety of frailty measures and the presence of co‐morbidities with end organ damage are important contributing factors to the decision for lung transplant listing.^205^

Outcomes after lung transplantation are less than ideal, with a median survival post lung transplantation for IPF internationally of 4.5 years, lower than that for cystic fibrosis and COPD (7.8 and 5.4 years, respectively).^206^ However, outcomes in Australia are better with combined Australian transplant centre data suggesting a median survival post lung transplantation in excess of 6 years for recipients with IPF.^207^ The reasons for the inferior survival for transplantation for IPF include the age of the recipients, the presence of co‐morbidities and the more frequent use of single lung transplantation. However, a diagnosis of IPF itself is also an independent risk factor for mortality.^201^

Unlike suppurative lung diseases, IPF patients are eligible for single, as well as double lung transplants. Bilateral lung transplantation is associated with superior long‐term post‐transplant outcomes.^201^ The choice is dependent on multiple factors including: the availability of potentially suitable donor lungs (vs. the anticipated waitlist mortality), and organ utility (two recipients can be transplanted from the same donor). Waitlist time is particularly prolonged for recipients who are smaller, who are blood group O and who are sensitized. In those with a small chest cavity, a larger single lung transplant may be possible, potentially expanding the donor pool.

Anti‐fibrotic therapy may theoretically impair wound healing, and the product information for both available anti‐fibrotic agents includes recommendations to withhold therapy around surgery. This has led to concerns about the safety of anti‐fibrotic therapy in IPF patients awaiting lung transplantation. Withdrawing anti‐fibrotic therapy pre‐transplant risks more rapid disease progression, acute exacerbation and death before transplant. There already exists a small but real risk of bronchial dehiscence after transplantation. Several small studies have now been published which provide data to guide decision‐making around the prescription of anti‐fibrotic therapy whilst awaiting transplantation. In an Australian lung transplant cohort, 40 (17.7%) of 226 were receiving anti‐fibrotics at the time of transplantation.^207^ In this cohort, there were seven episodes of anastomotic dehiscence, with overall incidence rates of 7.5% and 2.2% in the anti‐fibrotic and control groups, respectively (*p* = 0.08). In a small Japanese cohort (*n* = 25), patients awaiting lung transplantation who were receiving pirfenidone experienced less lung function decline and improved clinical condition at the time of transplantation as assessed by the Lung Allocation Score. There was no increase in post‐operative complications in the pirfenidone group, although the study was underpowered to detect differences.^208^ In a recently published study across nine United States transplant centres, there were 11 (5.2%) anastomotic and 12 (5.7%) sternal dehiscence events among 211 lung transplant recipients taking anti‐fibrotic therapy within five medication half‐lives (~2 days for nintedanib and 1 day for pirfenidone) of their surgery.^209^ There were no dehiscence events in the 86 recipients who ceased anti‐fibrotic therapy longer than five half‐lives before their surgery. There was no difference between groups in length of hospital stay or survival to discharge. Based on the above data, the decision to continue anti‐fibrotic therapy after lung transplant listing, should be individualized, taking in account the lung transplant centres preferences and the expected waitlist time. For example, an urgently listed individual with an anticipated waitlist time of weeks has probably very little to gain from continued anti‐fibrotic therapy, whereas an individual with unfavourable parameters to donor organ allocation and an anticipated wait of many months and potentially beyond a year, may have much to gain from continued therapy.

There has been an increasing recognition of the role of genetics in conferring IPF risk, with particular focus on mutations in telomere maintenance genes and their association with IPF and other ILDs. Patients with short‐telomere related pulmonary fibrosis are frequently younger, and experience a more aggressive disease course, and so are over‐represented in ILD transplant cohorts, with as many as 25% of patients with ILD in transplant centres having short telomeres.^210^ The syndromic nature of the so‐called telomeropathies means that post‐transplant complications, including extrapulmonary organ dysfunction^211^ (leukopenia and bone marrow failure, cirrhosis), infections and cancer, are more common in patients transplanted for short‐telomere related ILDs, with reduced overall and chronic lung allograft‐free survival in some, but not all studies.^210^, ^212^, ^213^, ^214^ A recent cohort study observed an increased risk of bronchial dehiscence in lung transplant recipients with short telomere‐related ILD.^215^ Lung transplantation is indicated for patients with short telomere‐related ILD, however, the risk of post‐transplant complications is increased, and post‐transplant survival may be reduced.

- *Lung transplantation is a viable option for selected patients with IPF/PPF and confers a survival benefit*.
- *Early referral for pre‐transplant assessment is essential, as it is difficult to accurately predict disease trajectory*.
- *Anti‐fibrotic therapy should be continued in most patients awaiting transplantation, so long as this is supported by the transplant centre*.

### Symptom management and palliative care

The goal of palliative care is to improve and maintain quality of life, for both patients and their caregivers. Palliative care encompasses both symptomatic care and comfort/end of life care and is separate to disease modifying therapies aimed at prolonging life. Palliative care is a holistic approach to the needs of patients, encompassing physical, psychological, social and spiritual aspects. Patient needs change throughout the course of the disease and the assessment of need is therefore an iterative and dynamic process, often taking into account differences in faith and culture.^216^ Early palliative care input benefits cancer patients by improving their quality of life and survival, as well as their family caregivers.^217^, ^218^, ^219^, ^220^ The European Respiratory Society published a Clinical Practice Guideline on palliative care for people with COPD and ILD in May 2023.^221^ They suggest that palliative care be initiated when there are unmet physical, psychological, social or spiritual/existential unmet needs. There is increasing recognition that early palliative care is beneficial for patients with chronic pulmonary diseases, given that patients still suffer from burdensome symptoms despite disease modifying therapies.^222^, ^223^, ^224^, ^225^, ^226^ This is particularly pertinent to IPF and PPF, where patients are faced with a significantly shortened survival, inevitable disease progression in an unpredictable fashion, and worsening symptoms despite anti‐fibrotic therapy. However, despite having a similar if not worse prognosis compared to many cancers, palliative care is generally offered late, if at all, to patients with IPF and PPF.^227^, ^228^, ^229^, ^230^ This is largely driven by the misconception that palliative care consists solely of end‐of‐life care, not only by the patients and their families, but also by health professionals.^231^, ^232^, ^233^ The negative connotations associated with the term is another significant barrier. When the term ‘supportive care’ was substituted for ‘palliative care’, clinicians referred patients earlier,^234^ and patients reported better understanding, more favourable impressions and higher future perceived need for ‘symptomatic care’.^235^ In IPF and PPF, symptomatic care should be initiated early and can aid in the delivery of bad news. It can be used to provide reassurance that holistic support will be provided throughout the disease course. This fosters an environment of hope despite worsening of disease and loss of quality of life. Advanced care planning (ACP) is another important aspect of palliative care and should be raised when patients are relatively well so that they are able to discuss their wishes with friends and family.

Breathlessness, cough and fatigue in IPF worsen despite treatment, and are associated with significant reductions in quality of life.^216^, ^236^ There are limited data for the use of pharmacological therapies for breathlessness in IPF and PPF. There is low quality evidence for the use of oral/ systemic opioids^237^, ^238^ and no evidence for the use of benzodiazepines.^239^, ^240^ In a small, randomized placebo‐controlled trial of 36 participants with fibrotic ILD, dyspnoea was reduced from baseline with four daily doses of 5 mg oral morphine, however, the difference compared to placebo was not statistically significant.^241^ A recent, large, randomized trial of regular, sustained‐release morphine in people with chronic dyspnoea, failed to show a benefit on dyspnoea measured on a visual analogue scale after 7 days of treatment.^242^ There was a reduced requirement for rescue immediate release morphine in the intervention arm.^242^ However, the majority of participants were breathless due to COPD or cancer, therefore, the relevance to patients with ILD is uncertain. The role of mirtazapine, an antidepressant, in relieving breathlessness is currently being explored.^243^, ^244^ The optimal approach to the pharmacologic management of dyspnoea in people with IPF and PPF is not known, however, data on the low doses of opioids utilized in this circumstance does not reveal any concern for respiratory depression.^237^ Non‐pharmacological approaches appear promising. In a three‐month pilot study of the hand‐held fan in patients with fibrotic lung disease, there was high acceptance of the therapy with some patients reporting benefit.^245^ The delivery of an integrated ‘breathlessness service’ has been shown to improve breathlessness as well as survival in patients with advanced respiratory disease.^223^, ^246^

Cough is difficult to manage in IPF and PPF with no uniformly effective treatments. Aside from excluding other causes of cough, oral steroids, cough suppressants and gabapentin might be effective in some patients and may be used empirically.^247^ Thalidomide was reported to be promising in one small study but was associated with significant side effects.^248^ In an observational study, pirfenidone was reported to improve cough in some patients.^13^ In a safety and efficacy study of inhaled sodium cromoglycate, patients reported a 31% reduction in cough.^249^ A recent small crossover trial of the opioid agonist nalbuphine reduced cough frequency during the 22 day treatment period.^250^

Fatigue in IPF is common and under‐recognized, and stems from multiple contributors, including physical deconditioning, poor sleep, comorbid conditions, hypoxia, dyspnoea, depression and anxiety.^251^ Effective management is likely to require a multifaceted approach. In addition to addressing physical symptoms, it is important to confront key psychological issues and comorbidities that detract further from quality of life.^216^, ^236^ It is important to stress that supportive care and lung transplantation are not mutually exclusive and that patients awaiting lung transplantation should not be deprived of this vital aspect of their care.

The best model of delivering palliative care to patients with IPF and PPF is yet to be established. Palliative care is traditionally delivered by palliative specialist physicians and nurses. Providing education and information about IPF and PPF to palliative care teams is crucial. Upskilling and training of ILD/chronic disease teams in the provision of palliative care should also be improved. Evidence demonstrates that a multi‐disciplinary approach to the provision of palliative care improves uptake of advanced care planning and certain approaches may also improve quality of life.^223^, ^246^, ^252^, ^253^ There is increasing evidence that ILD nurse‐led palliative care models are feasible and effective in supporting both the patients and their care givers.^254^, ^255^

- *Palliative care encompasses both symptomatic care and comfort/end of life care and should be considered in parallel to disease modifying pharmacotherapy and lung transplant evaluation*.

### Psychosocial/family support

The emotional impact of living with IPF and PPF can be significant for patients and their families. Not only do patients describe difficulties managing dyspnoea, cough, fatigue and other physical symptoms; they and their families also struggle with the emotional burden that a diagnosis brings. Traditionally, many patients report difficulty accessing accurate and up to date quality information about their condition and their treatment options, some also report that their treating specialist is hesitant to discuss prognosis.^256^ As the disease progresses, the loss of independence, greater reliance on others and loss of previous life roles, can elicit strong emotional distress.^257^, ^258^ Whilst emotional distress is a normal and expected reaction and not necessarily an indication that the patient is suffering from clinical anxiety or depression, they should, ideally be offered supportive psychotherapy at these times.^258^, ^259^

It is important that clinicians monitor patients for symptoms of anxiety and depression, which will appear more pervasive than emotional distress, especially as the disease progresses. Utilizing screening tools such as the Hospital Anxiety and Depression Scale (HADS) is one such way to monitor. However, if patients score highly on a screening tool, they should undergo a clinical interview to confirm a diagnosis of anxiety and/or depression or be referred to a clinical psychologist for assessment and therapy. An Australian survey of 124 people with ILD using the Hospital Anxiety and Depression Scale (HADS) identified anxiety and depression in 31% and 23%, respectively, which were clinically significant in 12% and 7%, respectively.^260^ The severity of dyspnoea and the presence of comorbidities were significant contributors to anxiety and depression. Similar data has been obtained through analysis of the Australian IPF Registry, which added cough severity as another significant contributor.^261^ A higher HADS has been associated with reduced physical activity in people with IPF.^262^ There are no studies evaluating the efficacy of anti‐depressant pharmacotherapies specifically in people with ILD, however, they are an important consideration in people with clinical depression, after taking into account drug interactions (e.g., fluvoxamine greatly increases levels of pirfenidone).

Anxiety and/or depression should not be confused with a grief reaction. Preparatory and or anticipatory grief will be experienced by many patients with IPF or PPF and can often present like depression.^263^ A grief reaction, however, is a normal and natural reaction to a diagnosis of IPF or PPF and may re‐occur at various points of the disease trajectory.^258^ Differentiating between grief and depression or anxiety can be difficult, however, asking the patient if the emotions come in waves (grief) or are there most of the time (depression) is a good starting point.^264^ Ideally, patients and their caregivers should be offered supportive psychotherapy to help them manage difficult emotions.^258^

Research shows that during these times patients and their families often report inadequate emotional support, both of a formal nature and from their wider support system.^182^, ^265^, ^266^, ^267^, ^268^, ^269^ As IPF is not a disease that has wide community recognition, patients frequently report difficulty in explaining their disease to family and friends.^182^ Overwhelmingly, patients want their family involved in discussions about treatment, disease progression and prognosis and also want resources to acknowledge the substantial emotional impact on not only patient, but also their family.

A variety of models for patient and family support have been evaluated. A nurse‐led model including support for patients and caregivers, education and symptom management was tested in 136 patient/caregiver dyads in an American expert centre. Improvements were seen in knowledge and advance care planning completion in patients and in knowledge, disease preparedness and confidence in caregivers.^255^ However, only half of those who were eligible agreed to participate in the program, which was delivered in person at clinic visits. IPF Care was another nurse‐led, industry‐sponsored education and support program for patients receiving pirfenidone in Europe, delivered via regular telephone calls and home visits, along with individually tailored information booklets.^270^ Retention in the program was 71% over 18 months and patient‐reported satisfaction was high.^270^ ILD‐specific support groups are another resource for many patients, where they can share experiences and coping skills with others in a similar situation.^271^ Consumer advocacy groups can also play an important role through representing the needs of people with ILD in the broader community.^182^, ^256^, ^266^, ^267^, ^268^

Locally, the Lung Foundation Australia and Centre of Research Excellence for Pulmonary Fibrosis (CRE‐PF) provide patients with ILD and their families with a range of resources for education, psychosocial support and advocacy. These include disease‐specific educational materials, webinars for patients and caregivers; a peer support program that connects patients living with IPF via telephone or web portal to share experiences, knowledge and support, regular scientific conferences that include a consumer stream enabling access to the most up‐to‐date information, and public advocacy for evidence‐based care and research to improve IPF and PPF outcomes. Further information can be found on the Lung Foundation Australia (Pulmonary Fibrosis Overview‐Lung Foundation Australia) and CRE‐PF websites (Centre Of Research Excellence In Pulmonary Fibrosis|Medical (cre-pf.org.au)).^272^ Many tertiary referral centres for ILD in Australia and New Zealand also provide extensive support to patients and families through their multidisciplinary teams, particularly ILD specialist nurses and clinic coordinators. Despite a growing acknowledgement that the patient and their family's psychological health is a very important factor in morbidity, mortality and compliance to treatment, most multi‐disciplinary teams do not have a psychologist as part of that team.^256^ In Australia and New Zealand, many patients and their families are referred privately for psychological therapy, however, financial considerations and high general demand on such resources may impact access.

- *The emotional impact of an IPF or PPF diagnosis is significant for both patients and their families/caregivers and is impacted upon by their individual cultural and social circumstances*.
- *Consider involving families/caregivers in discussions around diagnosis, treatment and prognosis*.
- *Consider the psychological impact of IPF and PPF routinely, and refer for specialist psychological support when indicated*.
- *Experiencing emotional distress by patients and their families/caregivers at various times throughout the disease course is normal, natural and to be expected. Psychological support should be offered to help normalize this reaction*.
- *Patients and families/caregivers should be provided with access to support groups and up to date educational material*.

## PRECISION MEDICINE AND GENETICS

Current evidence shows that genetic variants increasingly account for a significant proportion of risk for both familial (meaning two or more first‐degree relatives affected) and sporadic IPF.^273^ A family history of ILD is often present in cases of IPF, seen in 13% of participants in the Australian IPF Registry,^274^ and in up to 20% in other series.^275^ Telomere shortening secondary to mutations in telomere related genes are the most common genetic associations in families with pulmonary fibrosis and are typically inherited in an autosomal dominant pattern with incomplete penetrance.^276^ Additionally, telomere shortening is identified in up to 25% of sporadic IPF cases and associates with worse prognosis.^277^, ^278^, ^279^, ^280^ While measurement of telomere length is not routinely available, certain phenotypic features may be utilized to heighten suspicion (Table 6).^281^

**TABLE 6:** Features suggestive of shortened telomeres in a patient with pulmonary fibrosis.

Positive family history of ILD
Age of diagnosis under 50 years
Nail dystrophy
Premature hair greying (before 25 years)
Personal or family history of full blood count abnormalities (macrocytosis, thrombocytopenia)
Personal or family history of unexplained liver disease

Abbreviation: ILD, interstitial lung disease.

Evidence suggests those IPF patients with shortened telomeres are particularly susceptible to the harmful effects of immunosuppression. The PANTHER study in 2012 resulted in a major shift in the management of IPF.^282^ A recent analysis which included participants in the PANTHER study, has demonstrated that much of the harm attributed to the combination of *N*‐acetylcysteine, azathioprine and prednisolone, was experienced by the subset of patients with telomere lengths less than the tenth centile.^283^ This group of patients is frequently characterized by the presence of atypical radiology, which might be classified as non‐IPF. Trials of immunosuppression should be approached with caution in patients with ILD who might have shortened telomeres. Additionally this group of patients typically experience a more aggressive disease trajectory,^277^, ^278^, ^279^, ^280^, ^281^ and therefore, transplantation referral should be considered early. Importantly, shortened telomeres are not a contraindication to transplant, but do factor into transplant evaluation.^214^ Evidence is supportive of the efficacy of anti‐fibrotics for this group of patients.^284^ Therapies directly targeting the telomere apparatus are not yet available, however, there is active investigation into targeted therapies (NCT04638517).^285^, ^286^

Despite conflicting results across trials to date, there remains significant interest in the potential role of *N*‐acetylcysteine. Since the last position statement, a Japanese trial of nebulised *N*‐acetylcysteine demonstrated no benefit when added to pirfenidone.^287^ Notably, the frequency of the TT genotype of the TOLLIP rs3750920 gene which is potentially predictive of a favourable response to *N*‐acetylcysteine is known to be very low in the Japanese population.^288^ The PRECISIONS pharmacogenomic trial (NCT04300920) is currently recruiting to definitively evaluate the role of *N*‐acetylcysteine after selection for TOLLIP rs3750920 TT genotype. It is highly likely that the future treatment of IPF/PPF will be heavily influenced by genomic factors.

Interstitial lung abnormalities were identified in 23% of asymptomatic first‐degree relatives of patients with familial pulmonary fibrosis in a prospective, longitudinal study.^289^ Their presence occurred 7 years earlier than the average age of the affected family member at clinical diagnosis. Progression of these interstitial abnormalities at 5‐year follow‐up occurred in almost two thirds of patients. Progression of these interstitial abnormalities was associated with the presence of shortened telomeres and the MUC5B risk allele. A subsequent study suggests that the risk of ILD in asymptomatic relatives might extend beyond familial pulmonary fibrosis to the relatives of sporadic IPF cases.^290^ These data suggest that screening for ILD in asymptomatic relatives may enable earlier recognition and disease modifying intervention. Participants who underwent screening generally did not report negative psychological consequences or regret.^291^ While it is premature to recommend screening asymptomatic relatives of patients with IPF, in patients with a stronger suspicion for a familial/genetic aetiology, consideration should be given to formal clinical genetics' counselling and evaluation. Knowledge of a person's risk may lead to vigilance around lung health (e.g., smoking cessation) and enable a heightened awareness of primary care physicians when such an individual presents with respiratory symptoms.

- *Consider a genetic cause of IPF in patients with a family history of pulmonary fibrosis and/or early age of IPF diagnosis. Such patients may experience rapid deterioration and early referral to a lung transplant unit should be considered*.
- *Clinicians should be vigilant for phenotypic features of shortened telomeres*.
- *IPF patients with shortened telomeres are at heightened risk of harm from immunosuppression. Caution should be exercised when considering immunosuppression in this patient group*.
- *Referral to a clinical geneticist should be considered for patients with familial ILD*.

## CONCLUSIONS AND FUTURE CONSIDERATIONS

This 2023 Treatment of Idiopathic Pulmonary Fibrosis and Progressive Pulmonary Fibrosis Position Statement provides an important update to the multi‐disciplinary management of patients with IPF, and now also includes the PPF clinical behaviour entity. Treatment of IPF with anti‐fibrotic therapy is now well‐established and accepted practice. The use of nintedanib has broadened to include patients with PPF. For both IPF and PPF, it is critically important to consider other aspects of management outside of pharmacotherapy. Despite current limitations in the management of IPF and PPF, through a global collaborative effort and clinical trial endeavours, the future for this patient cohort looks promising. The management of ILD in the future is likely to become increasingly complex with multi‐dimensional pharmacological and non‐pharmacological therapy. Assessment of risk will improve through sophisticated‐omics methods and treatments will necessarily become increasingly individualized. We encourage clinicians involved in the management of these patients to strive to provide comprehensive care to improve their outcomes. The future for the next generation of patients with IPF and PPF is promising and through collaborative effort, achievable.

## CONFLICT OF INTEREST STATEMENT

John A. Mackintosh—reports speaker fees from Boehringer Ingelheim, Gregory Keir—reports advisory board, travel and speaker fees from Boehringer Ingelheim and Roche, Lauren K. Troy—reports speaker fees from Boehringer Ingelheim and Erbe Elektromedezin, and advisory board roles for Boehringer Ingelheim and Roche, Anne E. Holland—reports no conflicts of interest, Christopher Grainge—reports being an investigator for clinical trials sponsored by Boehringer Ingelheim, Syneos and Roche; and reports contractual basic laboratory science conducted on behalf of Boehringer Ingelheim, Daniel C. Chambers—reports speaker fees from Boehringer Ingelheim, Debra Sandford—reports no conflicts of interest, Helen E. Jo—reports no conflicts of interest, Ian Glaspole—reports advisory board fees from Boehringer Ingelheim and consulting fees from Amplia, Accendatech, Lassen, Tianli, Ad Alta and Avalyn, Margaret Wilsher—reports speaker fees from Boehringer Ingelheim and investigator role for trials sponsored by Boehringer Ingelheim, Syneos and Roche, Nicole S. L. Goh—reports no conflicts of interest, Paul N. Reynolds—reports no conflicts of interest, Sally Chapman—reports no conflicts of interest, Steven E. Mutsaers—reports no conflicts of interest, Sally de Boer—reports no conflicts of interest, Susanne Webster—reports no conflicts of interest, Yuben Moodley—reports no conflicts of interest, Tamera J. Corte—reports personal fees for speaking commitments, travel and advisory board membership from Boehringer Ingelheim, Roche, and Bristol Myers Squibb, Vicore, Bridge Therapeutics, and DevPro, and institutional fees for unrestricted grants from Boehringer Ingelheim, Roche, Galapagos, Biogen, Bristol Myers Squibb and Actelion. Christopher Grainge, Debra Sandford, Paul N. Reynolds, Yuben Moodley and Tamera J. Corte are Editorial Board members of *Respirology*—they were excluded from all editorial decision‐making related to the acceptance of this article for publication.
